# Supplementary material for: Multiscale modeling of host–pathogen interactions and mucociliary clearance during non-tuberculous mycobacterial pulmonary infection
Source: ArXiv. 2026 Sep 14:arXiv:2609.15584v1. Preprint. [Version 1] (PMC13596621)
Supplement: Supplementary file 1 [file NIHPP2609.15584V1-supplement-1.pdf]

# Supplementary Materials

## S1 Selection of Agent-Based Model (ABM) Simulations for Calibration

10 runs of the nontuberculous mycobacteria (NTM) airway infection ABM were selected to calibrate the partial differential equation (PDE) model. The 10 runs were chosen to capture the full range of ABM simulation results, with enrichment for edge cases. The plot below shows the final bacteria and macrophage counts for all ABM runs, with the 10 selected runs shown in red dots.

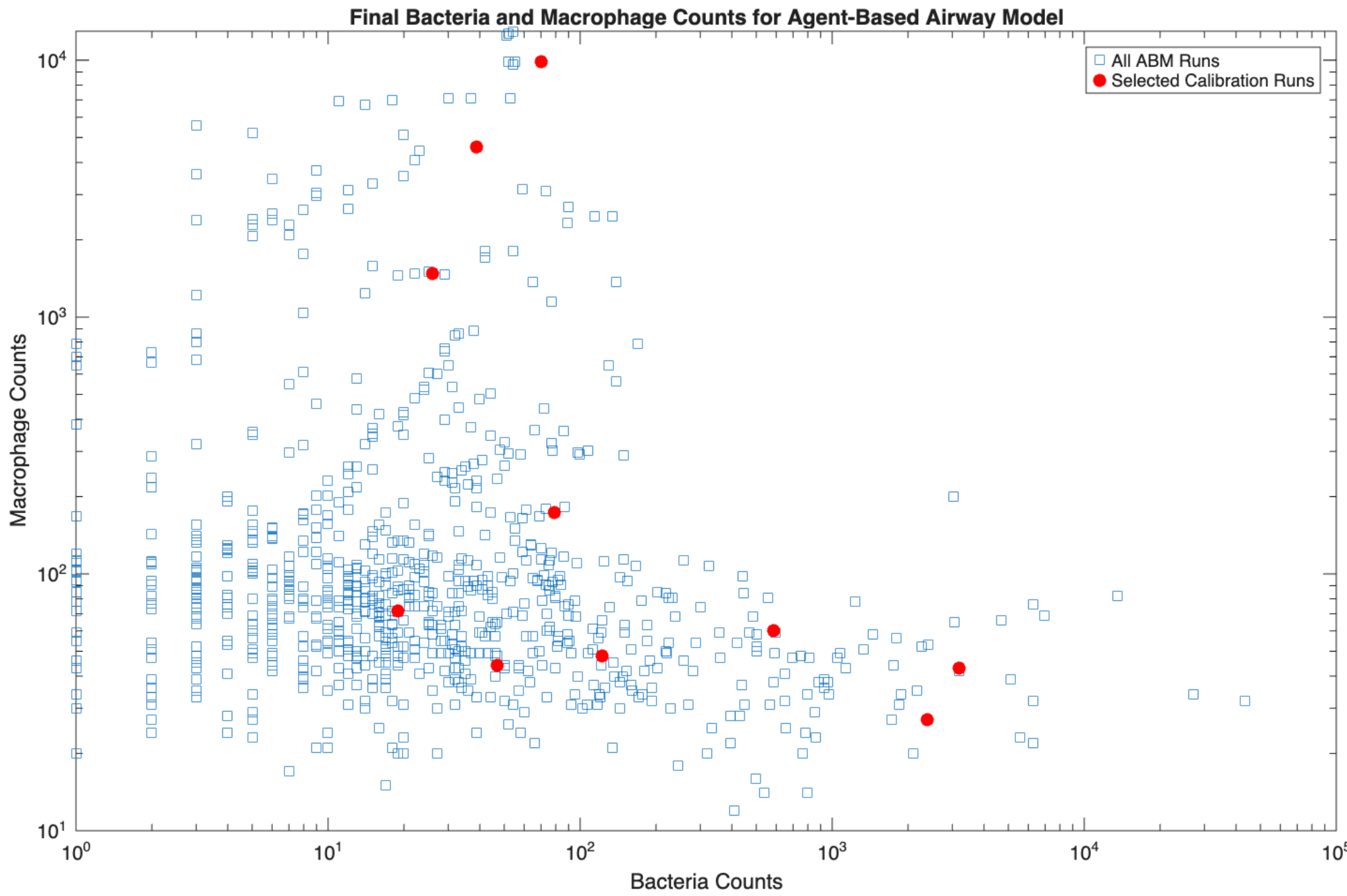


**Figure S1:** Final bacteria and macrophage counts from the nontuberculous mycobacteria (NTM) airway infection agent-based model. The runs selected for calibration of the partial differential equation model are shown in red.

## S2 ABM to Partial Differential Equation (PDE) Model Parameter Conversions

### S2.1 NTM Bacteria Death Rate $d_B$

In the ABM, infected macrophages may kill an intracellular NTM bacterium with a certain probability each 6-minute time step. The following method to convert this bacteria kill probability to a bacteria death rate was adapted from Jones et al. 2017 [23].

$$\begin{aligned} p(t) &= 1 - e^{-rt} \\ 1 - p(t) &= e^{-rt} \\ ln(1 - p(t)) &= -rt \\ r &= \frac{-ln(1 - p(t)}{t} \end{aligned}$$

where p(t) is the probability at time t.

For a 6-minute ABM timestep:

$$r = \frac{-ln(1 - \text{ABM bacteria kill probability})}{6 \text{ min}} \times \frac{60 \text{ min}}{1 \text{ hr}} \times \frac{24 \text{ hr}}{1 \text{ day}}$$
$$= -240ln(1 - \text{ABM bacteria kill probability})$$

To test the validity of this parameter conversion, we reran the 10 selected ABM simulations with no bacteria proliferation and compared the bacteria time course outputs with those from an ODE model of bacteria death, shown below.

$$\frac{dB}{dt} = -240ln(1 - \text{ABM bacteria kill probability}) \times B$$

where B is bacteria concentration in $g/cm^3$.

Figure S2 shows the ODE and ABM bacteria time courses for each of the 10 runs, demonstrating that the ODE model recapitulates the ABM results and supporting the validity of the bacteria death parameter conversion.

## S2.2 Homeostatic Macrophage Recruitment $\alpha_0$

In non-infection conditions, the number of newly recruited macrophages must counteract the number of macrophages that undergo apoptosis. Consider the following steady-state case

$$\frac{dM}{dt} = r_h - d_M M = 0$$

where $r_h$ is the macrophage recruitment at homeostasis and $d_M$ is the macrophage death rate. The number of newly recruited macrophages must counteract the concentration of macrophages lost. If a concentration of $M_0$ macrophages is initialized, then

$$d_M M_0 = \frac{\text{(Number of newly recruited macrophages)(Macrophage density)(Macrophage volume)}}{\text{ABM Simulation Volume}}$$

$$\text{Recruited macrophages per day} = \frac{d_M M_0 \text{(ABM Simulation Volume)}}{\text{(Macrophage density)(Macrophage volume)}}$$

In order to translate ABM cell agent data to the PDE model, we assume a well-mixed ABM simulation space. In the ABM, new macrophages are recruited at the bottom layer of the ABM simulation environment. Calculating the recruited macrophages per volume at the bottom layer of the ABM environment,

$$\frac{d_M M_0 \text{(ABM Simulation Volume)}}{\text{(Macrophage density)(Macrophage volume)(Simulation Bottom Layer Volume)}}$$

After converting to units of concentration of macrophages,

$$\begin{aligned}
&\frac{d_M M_0 \text{(ABM Simulation Volume)}}{\text{(Macrophage density)(Macrophage volume)(Simulation Bottom Layer Volume)}} \\
&\quad \times \text{(Macrophage Density)(Macrophage Volume)} \\
&= \frac{d_M M_0 \text{(ABM Simulation Volume)}}{\text{(Simulation Bottom Layer Volume)}} \\
&= \frac{d_M M_0 (0.2 \times 0.2 \times 0.006 \text{ cm}^3)}{0.2 \times 0.2 \times 0.002 \text{ cm}^3} \\
&= 3 \cdot d_M M_0 \text{ g/cm}^3 \cdot \text{day}
\end{aligned}$$

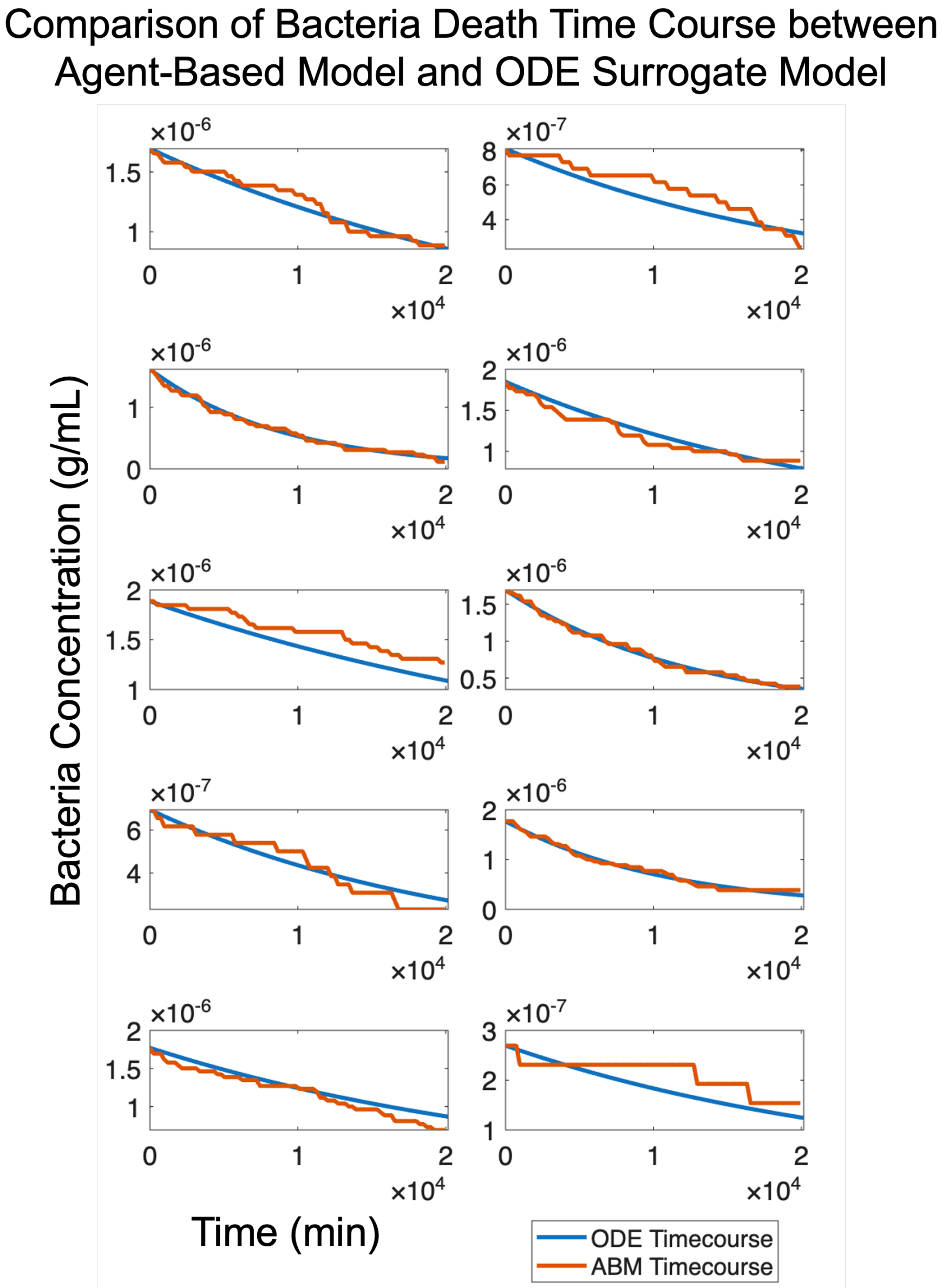


**Figure S2:** Bacteria concentration time courses between the ordinary differential equation bacteria death model and the adjusted agent-based model with no bacteria replication.

Assuming well-mixed conditions, this is equal to the concentration of macrophages per unit length of the ABM simulation environment, yielding the final conversion

$$3 \cdot d_M M_0 \text{ g/cm}^4 \cdot \text{day}$$

# S3 Units of model variables and parameters

**Table S1:** Variables and parameters in the PDE model and their units.

| Symbol | Description | Unit |
|---|---|---|
| $\boldsymbol{x}$ | Spatial coordinate | cm |
| $t$ | Time | day |
| $B(\boldsymbol{x}, t)$ | Bacterial density | $\mathrm{g\,cm^{-3}}$ |
| $M(\boldsymbol{x}, t)$ | Macrophage density | $\mathrm{g\,cm^{-3}}$ |
| $m(\boldsymbol{x}, t)$ | Mucus density | $\mathrm{g\,cm^{-3}}$ |
| $\boldsymbol{u}(\boldsymbol{x}, t)$ | Advection velocity | $\mathrm{cm\,day^{-1}}$ |
| $\lambda_B$ | Bacterial growth rate | $\mathrm{day^{-1}}$ |
| $K_B$ | Bacterial carrying capacity | $\mathrm{g\,cm^{-3}}$ |
| $K_{Bi}$ | Half-saturation bacterial density | $\mathrm{g\,cm^{-3}}$ |
| $d_B$ | Macrophage-mediated bacterial clearance rate | $\mathrm{cm^3\,g^{-1}\,day^{-1}}$ |
| $d_{Mb}$ | Maximum macrophage burst rate | $\mathrm{day^{-1}}$ |
| $d_M$ | Macrophage death rate | $\mathrm{day^{-1}}$ |
| $d_m$ | Mucus degradation rate | $\mathrm{day^{-1}}$ |
| $D_B(D_1, D_2)$ | Bacterial diffusion coefficients | $\mathrm{cm^2\,day^{-1}}$ |
| $D_M$ | Macrophage diffusion coefficient | $\mathrm{cm^2\,day^{-1}}$ |
| $D_m$ | Mucus diffusion coefficient | $\mathrm{cm^2\,day^{-1}}$ |
| $\delta$ | Chemotaxis coefficient | $\mathrm{cm^5\,g^{-1}\,day^{-1}}$ |
| $\gamma_B$ | Bacterial outflow coefficient | $\mathrm{cm\,day^{-1}}$ |
| $\gamma_M$ | Macrophage outflow coefficient | $\mathrm{cm\,day^{-1}}$ |
| $\gamma_m$ | Mucus outflow coefficient | $\mathrm{cm\,day^{-1}}$ |
| $\tau_B$ | Bacterial interface permeability coefficient | $\mathrm{cm^{-1}}$ |
| $\tau_M$ | Macrophage interface permeability coefficient | $\mathrm{cm^{-1}}$ |
| $M_{\mathrm{ref}}$ | Reference macrophage density | $\mathrm{g\,cm^{-3}}$ |
| $m_0$ | Reference mucus density | $\mathrm{g\,cm^{-3}}$ |
| $\alpha(B)$ | Macrophage recruitment coefficient | $\mathrm{cm\,day^{-1}}$ |
| $\alpha_0$ | Baseline macrophage recruitment coefficient | $\mathrm{cm\,day^{-1}}$ |
| $\alpha_1$ | Bacteria-dependent recruitment coefficient | $\mathrm{cm^4\,g^{-1}\,day^{-1}}$ |
| $\beta(MB)$ | Mucus production coefficient | $\mathrm{cm\,day^{-1}}$ |
| $\beta_0$ | Baseline mucus production coefficient | $\mathrm{cm\,day^{-1}}$ |
| $\beta_1$ | Inflammation-dependent mucus production coefficient | $\mathrm{cm^7\,g^{-2}\,day^{-1}}$ |
| $\eta$ | viscosity factor | Dimensionless |
| $\eta_0$ | Reference viscosity | Dimensionless |
| $d_{\mathrm{anti}}$ | Treatment-induced bacterial clearance rate | $\mathrm{day^{-1}}$ |
| $\zeta$ | Treatment-strength parameter | Dimensionless |

# S4 Calibration parameters

The model parameters selected for calibration are summarized in Table S2.

| | | | | | | |
|---|---|---|---|---|---|---|
| $D_B$ | $\lambda_B$ | $K_B$ | $K_{Bi}$ | $d_{Mb}$ | $d_B$ | $\tau_B$ |
| $D_M$ | $\delta$ | $d_M$ | $\tau_M$ | $M_0$ | $\alpha_0$ | $\alpha_1$ |

**Table S2:** List of model parameters subject to calibration.

## S5 Evolution Results Based on Calibration

This section presents the system evolution for different parameter sets. As in the main text, the results compare the ABM-derived data with the calibrated PDE simulations in the mucus region and the corresponding PDE predictions in the tissue region. The spatial evolution of the PDE system is shown at selected time points, both with and without the mucus velocity field, in Figures S3–S12.

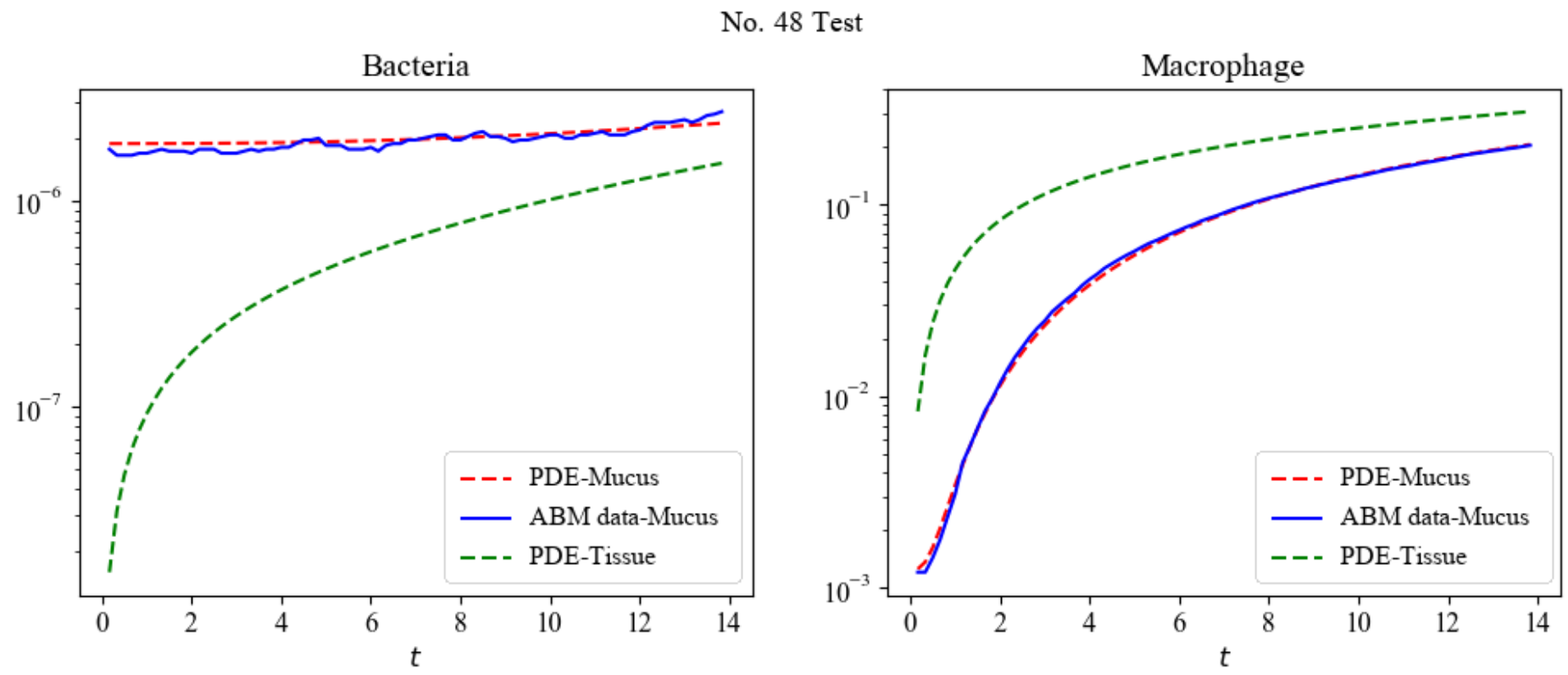


**(a)** Concentration fitting.

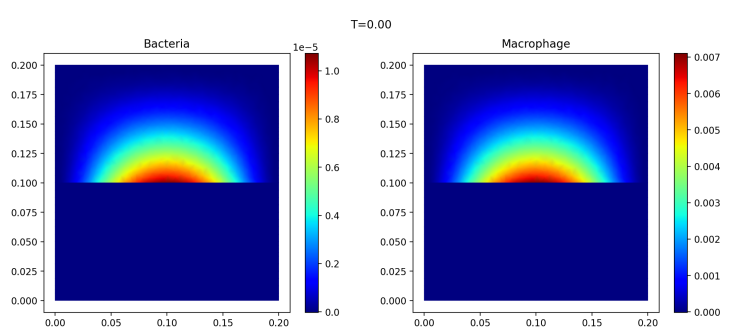

**(b)** No velocity, $t = 0$ day.

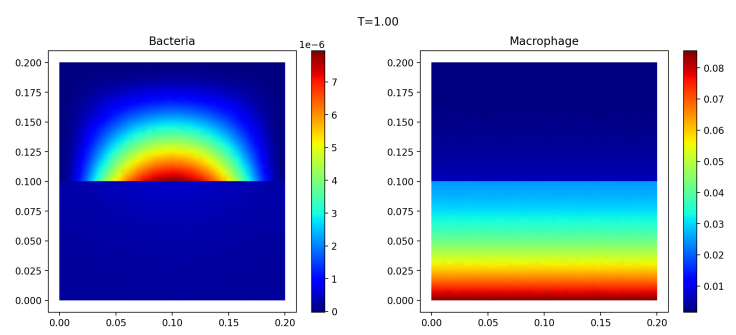

**(c)** No velocity, $t = 1$ day.

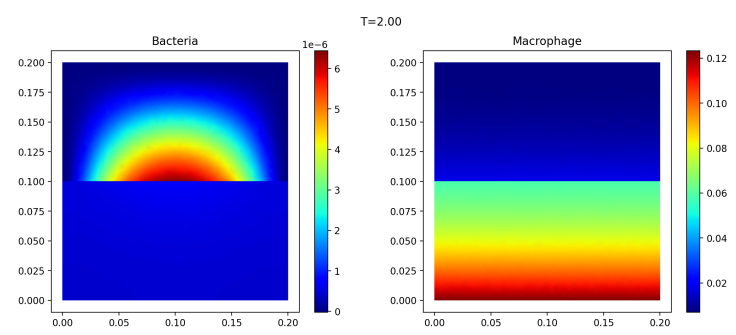

**(d)** No velocity, $t = 2$ day.

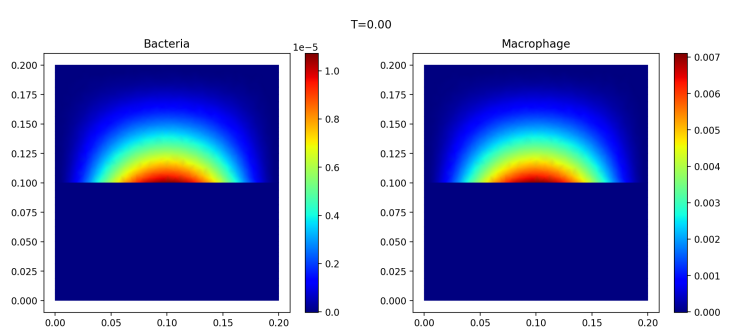

**(e)** With velocity, $t = 0$ day.

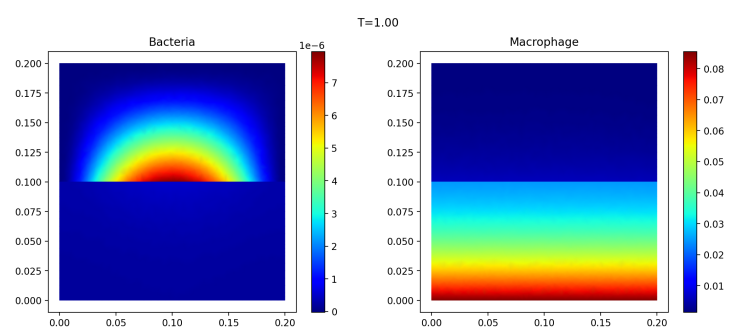

**(f)** With velocity, $t = 1$ day.

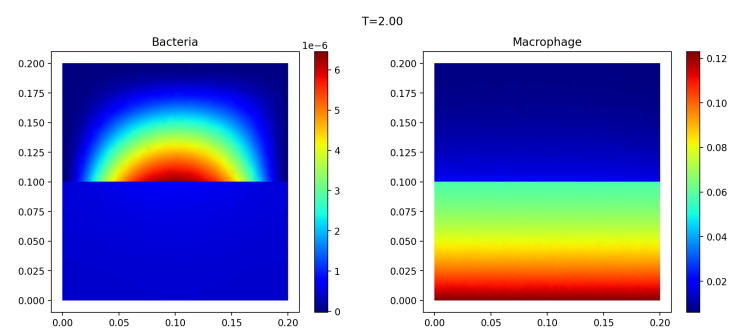

**(g)** With velocity, $t = 2$ day.

**Figure S3:** Evolution of the system for parameter set No. 48, with and without the mucus velocity field. (a) Concentration fitting for bacteria and macrophages, with calibration reference data, mucus-region simulation, and tissue-region simulation shown in each plot. (b–d) Spatial distributions without mucus velocity field driven at $t = 0$, 1, and 2 day. (e–g) Spatial distributions with the mucus velocity field driven at the same time points. Each spatial panel shows bacteria on the left and macrophages on the right.

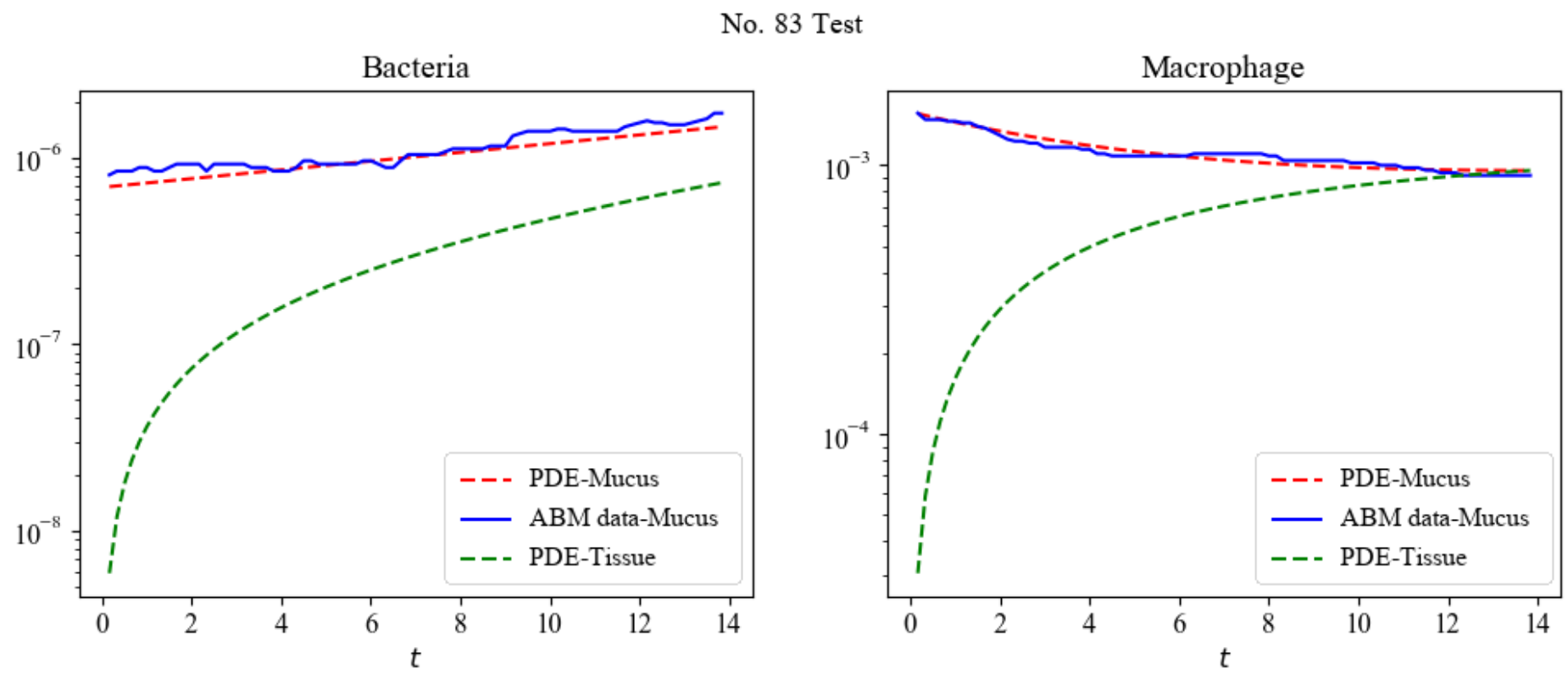


**(a)** Concentration fitting.

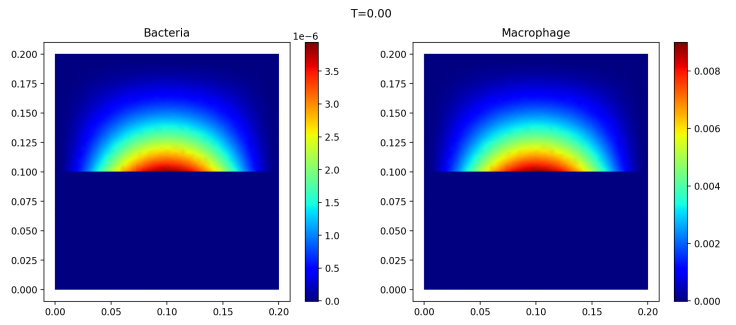


**(b)** No velocity, $t = 0$ day.

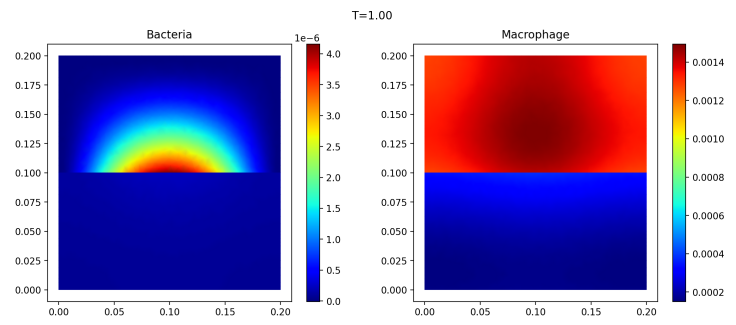


**(c)** No velocity, $t = 1$ day.

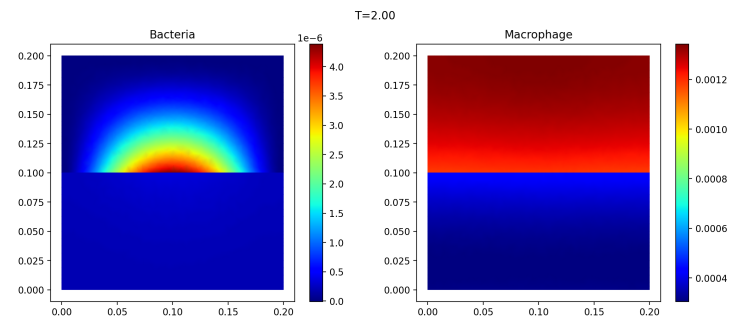


**(d)** No velocity, $t = 2$ day.

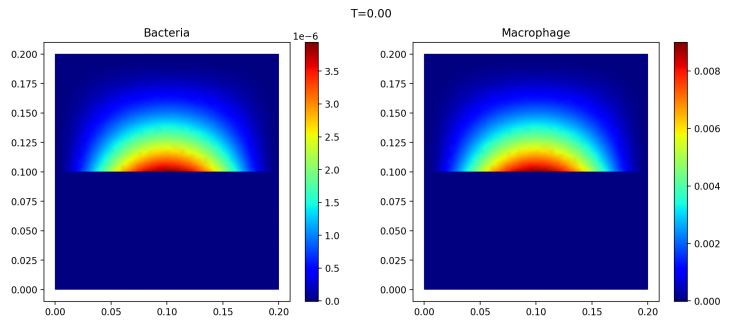


**(e)** With velocity, $t = 0$ day.

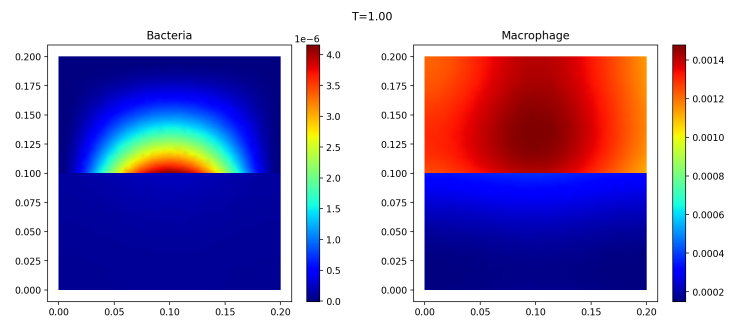


**(f)** With velocity, $t = 1$ day.

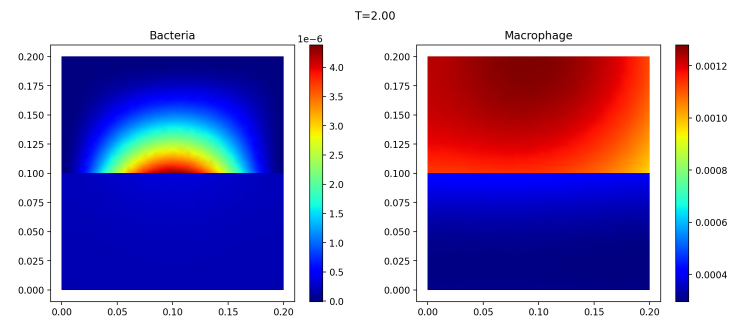


**(g)** With velocity, $t = 2$ day.

**Figure S4:** Evolution of the system for parameter set No. 83, with and without the mucus velocity field. (a) Concentration fitting for bacteria and macrophages, with calibration reference data, mucus-region simulation, and tissue-region simulation shown in each plot. (b–d) Spatial distributions without mucus velocity field driven at $t = 0$, 1, and 2 day. (e–g) Spatial distributions with the mucus velocity field driven at the same time points. Each spatial panel shows bacteria on the left and macrophages on the right.

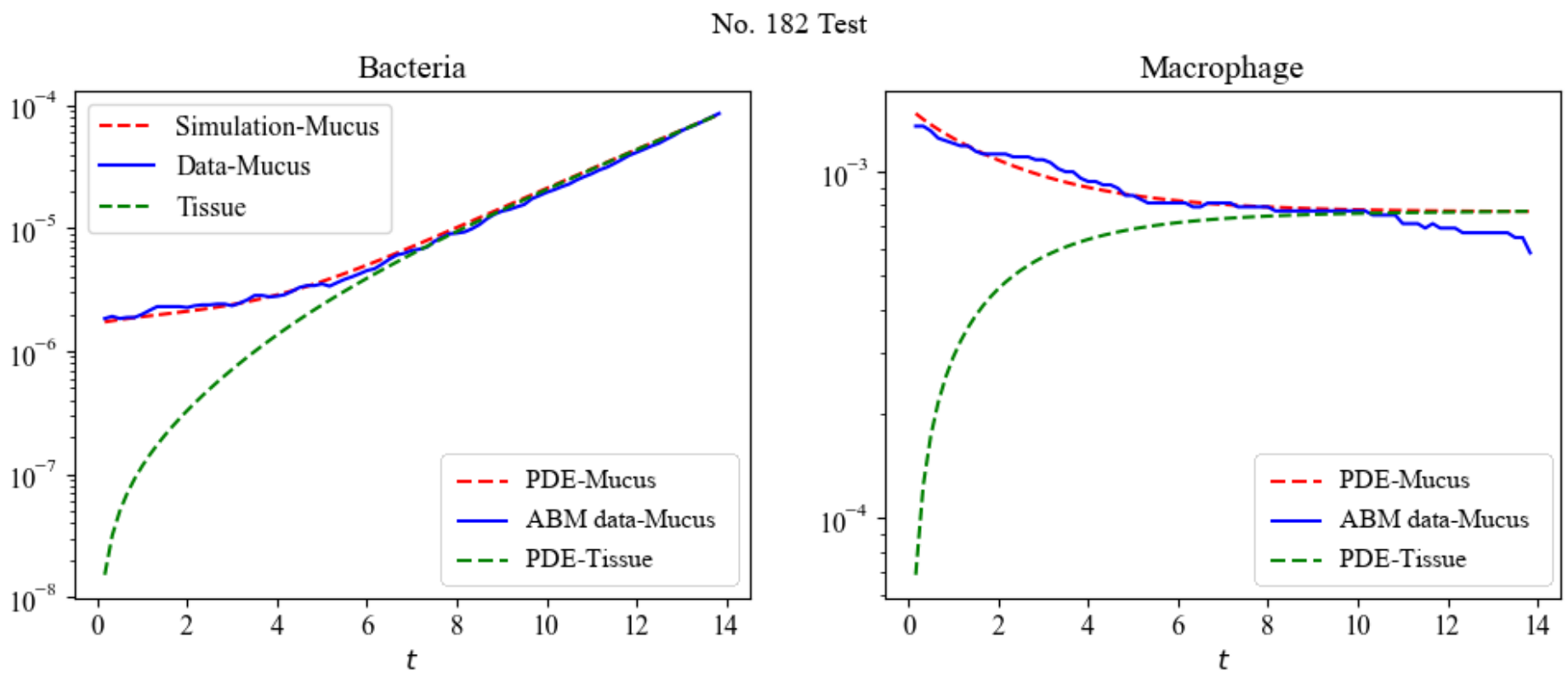


**(a)** Concentration fitting.

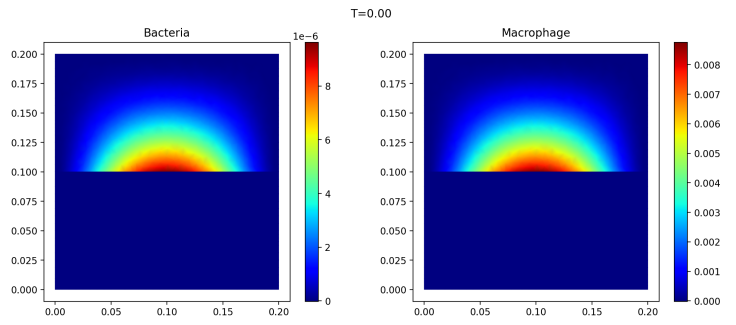


**(b)** No velocity, $t = 0$ day.

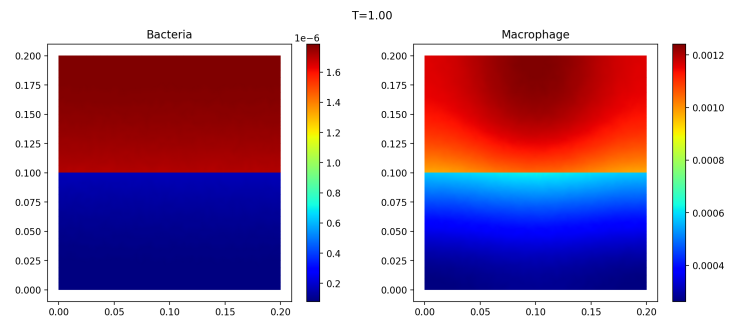


**(c)** No velocity, $t = 1$ day.

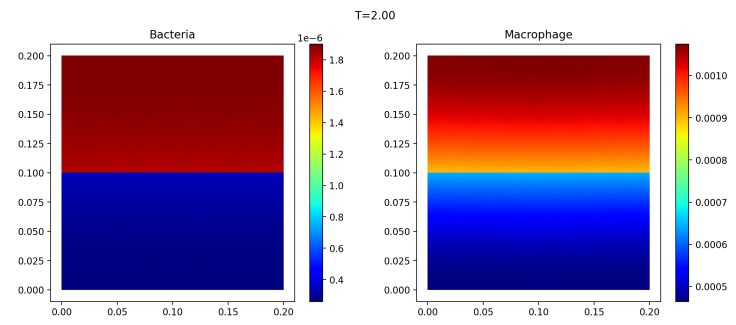


**(d)** No velocity, $t = 2$ day.

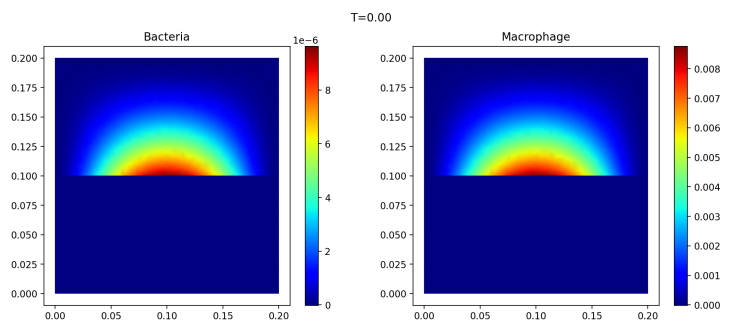


**(e)** With velocity, $t = 0$ day.

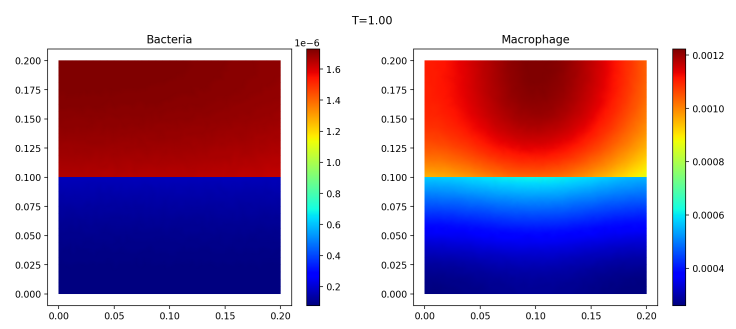


**(f)** With velocity, $t = 1$ day.

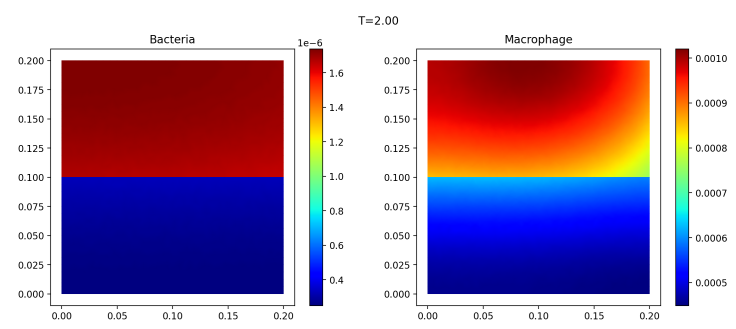


**(g)** With velocity, $t = 2$ day.

**Figure S5:** Evolution of the system for parameter set No. 182, with and without the mucus velocity field. (a) Concentration fitting for bacteria and macrophages, with calibration reference data, mucus-region simulation, and tissue-region simulation shown in each plot. (b–d) Spatial distributions without mucus velocity field driven at $t = 0$, 1, and 2 day. (e–g) Spatial distributions with the mucus velocity field driven at the same time points. Each spatial panel shows bacteria on the left and macrophages on the right.

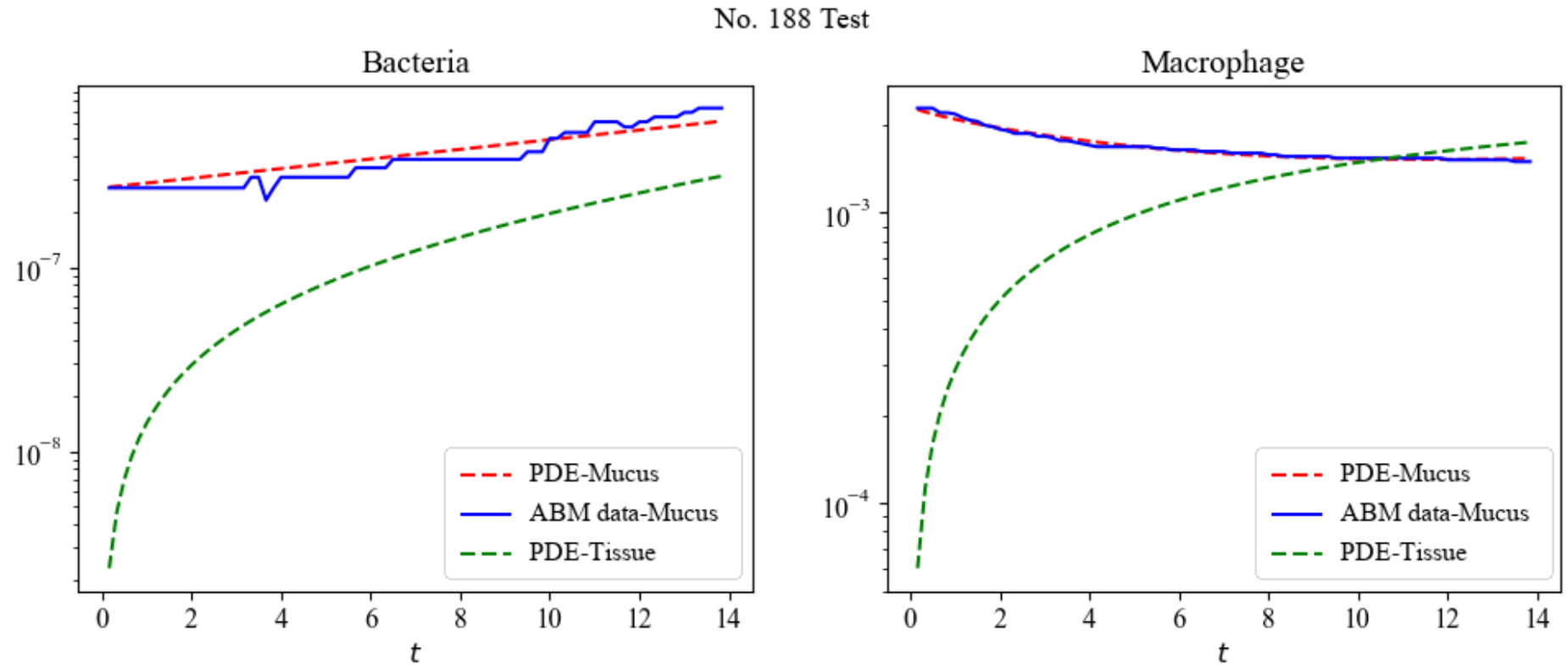


**(a)** Concentration fitting.

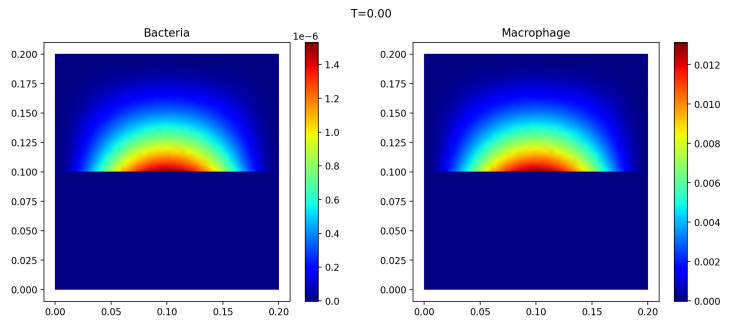


**(b)** No velocity, $t = 0$ day.

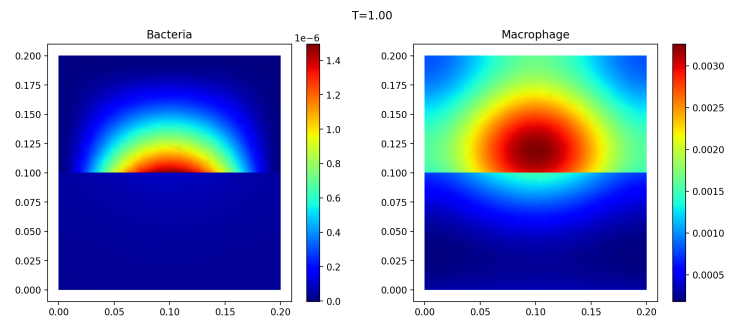


**(c)** No velocity, $t = 1$ day.

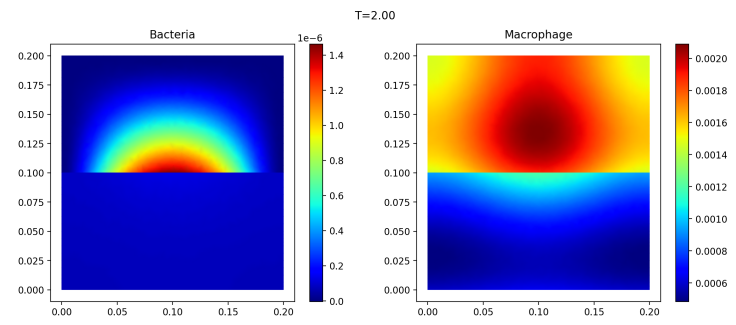


**(d)** No velocity, $t = 2$ day.

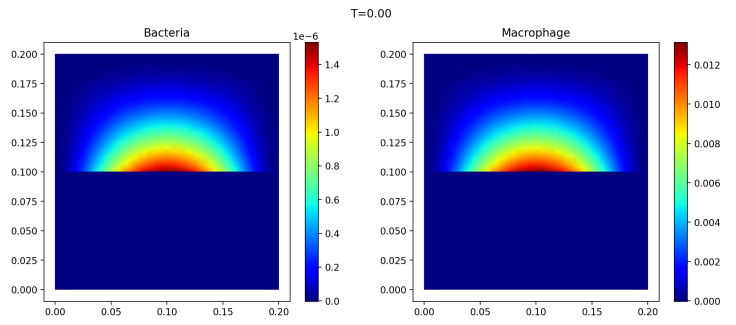


**(e)** With velocity, $t = 0$ day.

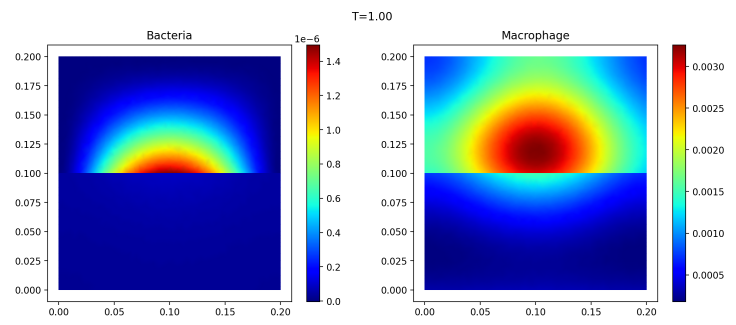


**(f)** With velocity, $t = 1$ day.

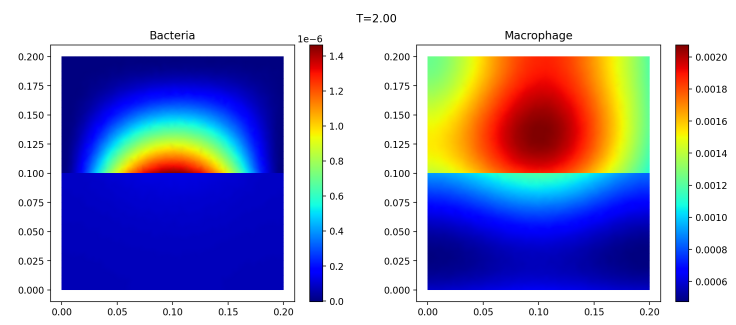


**(g)** With velocity, $t = 2$ day.

**Figure S6:** Evolution of the system for parameter set No. 188, with and without the mucus velocity field. (a) Concentration fitting for bacteria and macrophages, with calibration reference data, mucus-region simulation, and tissue-region simulation shown in each plot. (b–d) Spatial distributions without mucus velocity field driven at $t = 0$, 1, and 2 day. (e–g) Spatial distributions with the mucus velocity field driven at the same time points. Each spatial panel shows bacteria on the left and macrophages on the right.

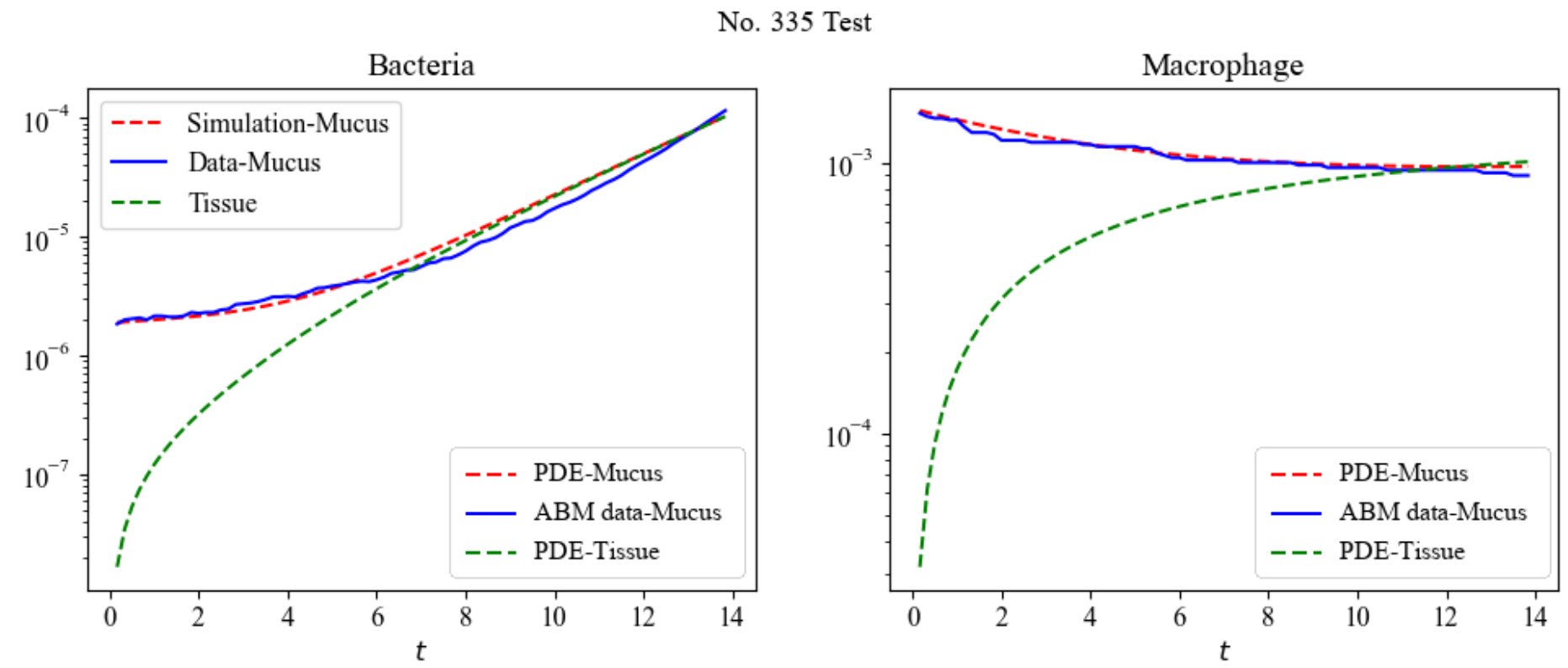


**(a)** Concentration fitting.

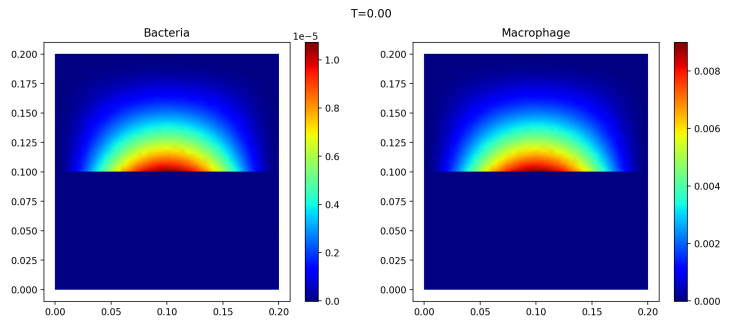

**(b)** No velocity, $t = 0$ day.

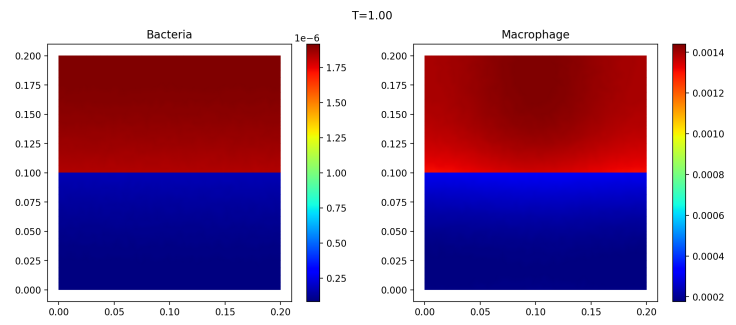

**(c)** No velocity, $t = 1$ day.

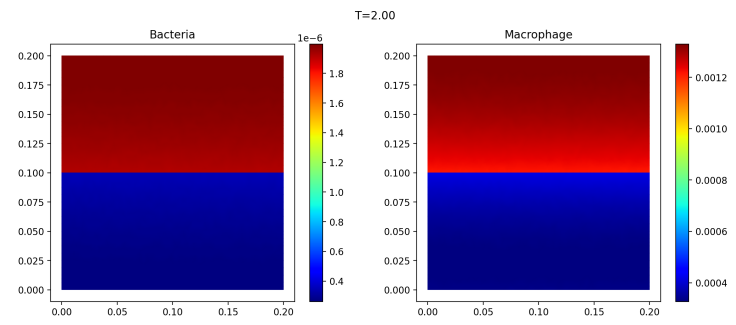

**(d)** No velocity, $t = 2$ day.

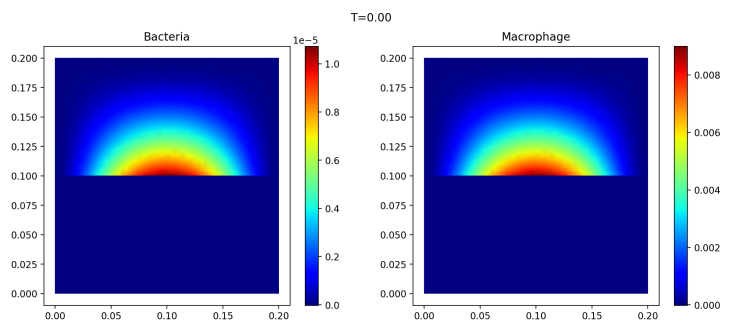

**(e)** With velocity, $t = 0$ day.

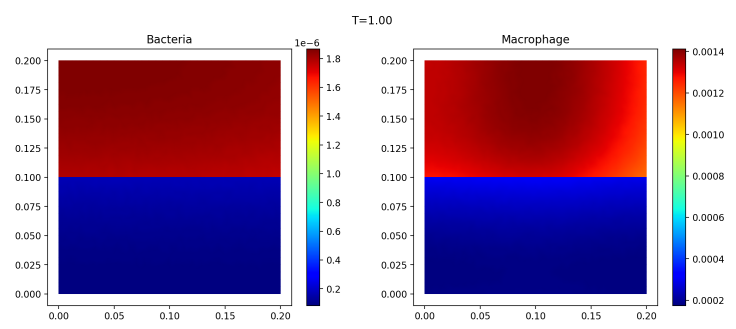

**(f)** With velocity, $t = 1$ day.

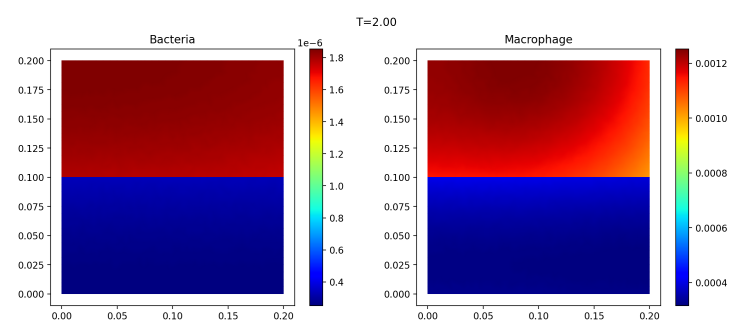

**(g)** With velocity, $t = 2$ day.

**Figure S7:** Evolution of the system for parameter set No. 335, with and without the mucus velocity field. (a) Concentration fitting for bacteria and macrophages, with calibration reference data, mucus-region simulation, and tissue-region simulation shown in each plot. (b–d) Spatial distributions without mucus velocity field driven at $t = 0$, 1, and 2 day. (e–g) Spatial distributions with the mucus velocity field driven at the same time points. Each spatial panel shows bacteria on the left and macrophages on the right.

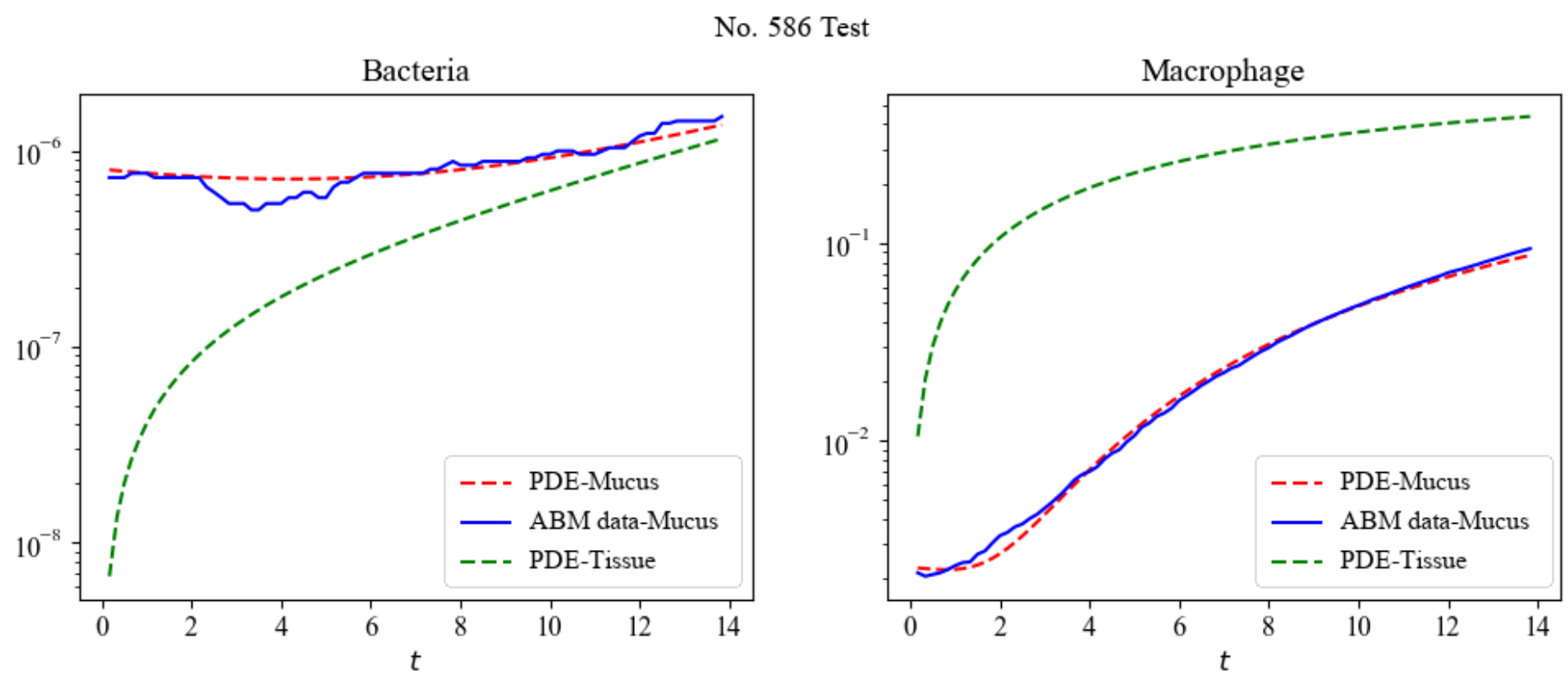


**(a)** Concentration fitting.

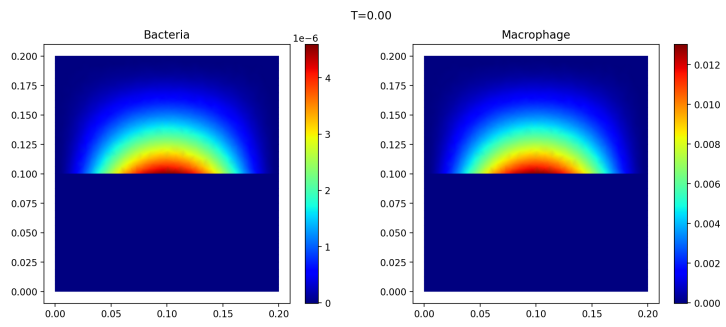


**(b)** No velocity, $t = 0$ day.

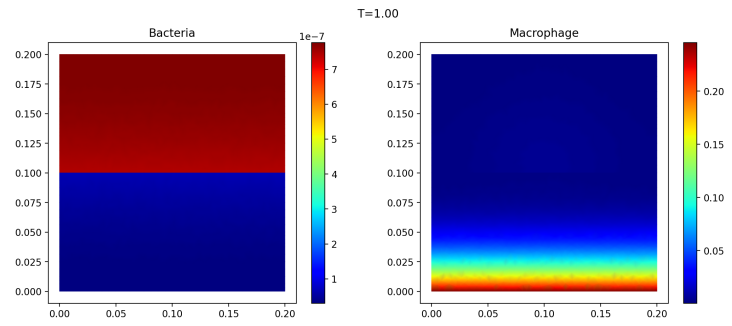


**(c)** No velocity, $t = 1$ day.

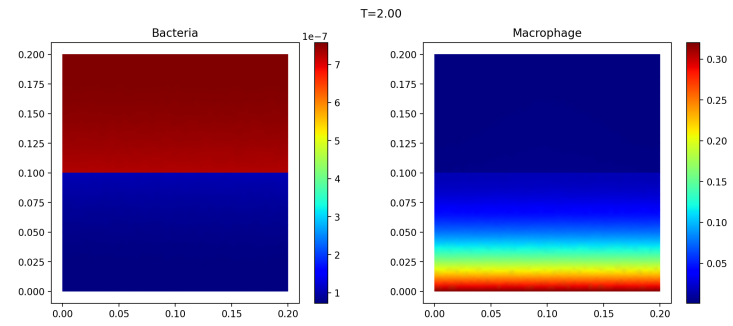


**(d)** No velocity, $t = 2$ day.

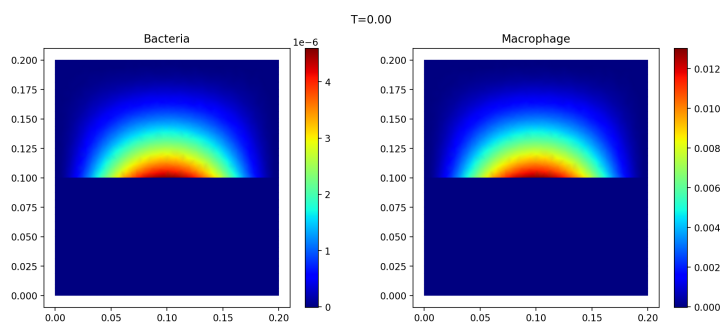


**(e)** With velocity, $t = 0$ day.

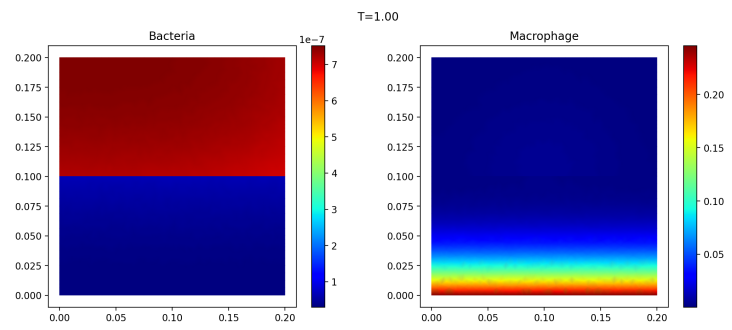


**(f)** With velocity, $t = 1$ day.

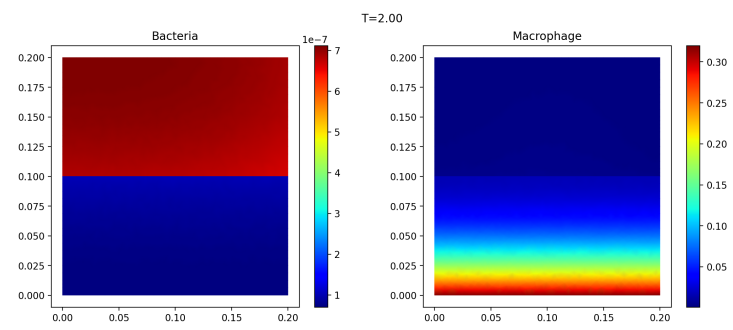


**(g)** With velocity, $t = 2$ day.

**Figure S8:** Evolution of the system for parameter set No. 586, with and without the mucus velocity field. (a) Concentration fitting for bacteria and macrophages, with calibration reference data, mucus-region simulation, and tissue-region simulation shown in each plot. (b–d) Spatial distributions without mucus velocity field driven at $t = 0$, 1, and 2 day. (e–g) Spatial distributions with the mucus velocity field driven at the same time points. Each spatial panel shows bacteria on the left and macrophages on the right.

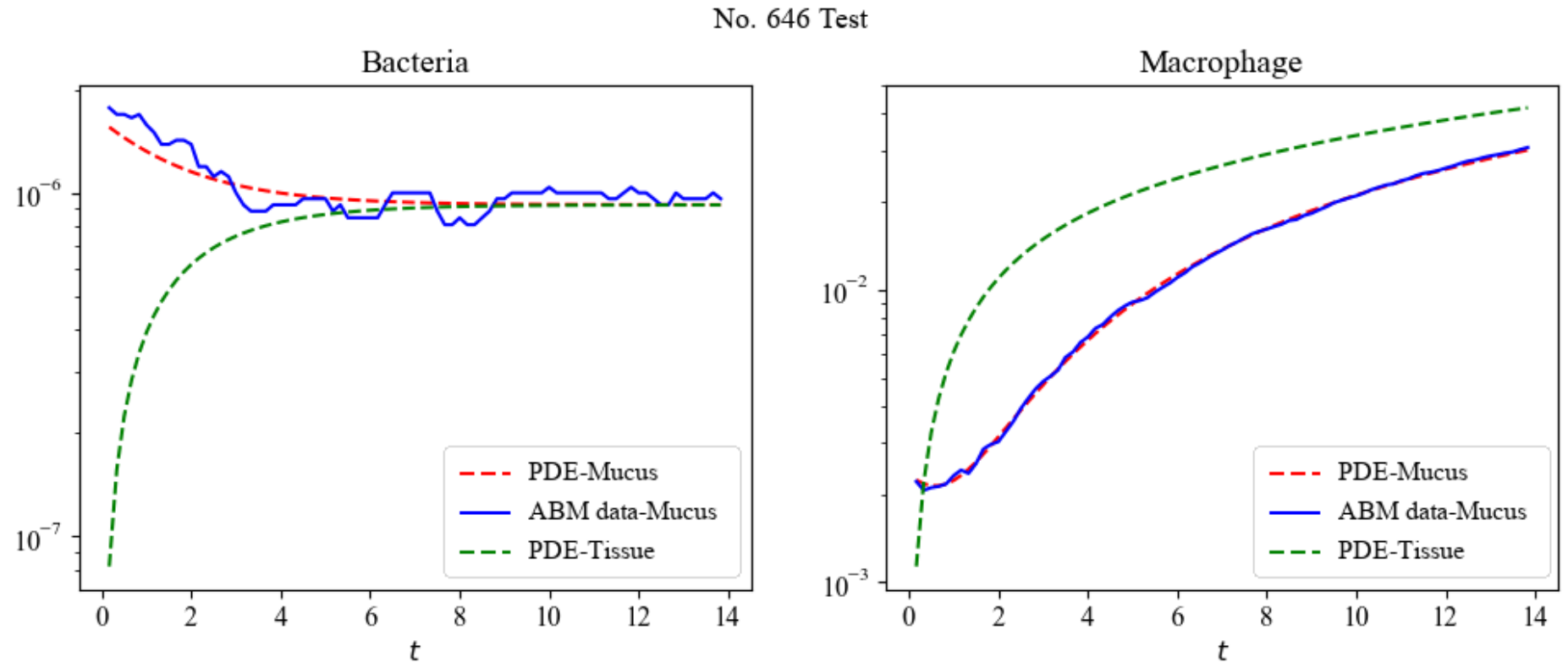


**(a)** Concentration fitting.

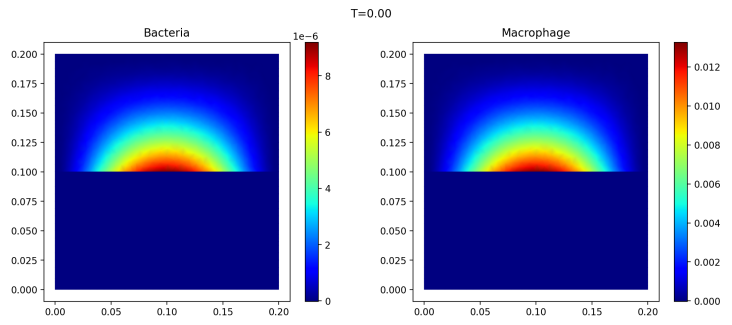


**(b)** No velocity, $t = 0$ day.

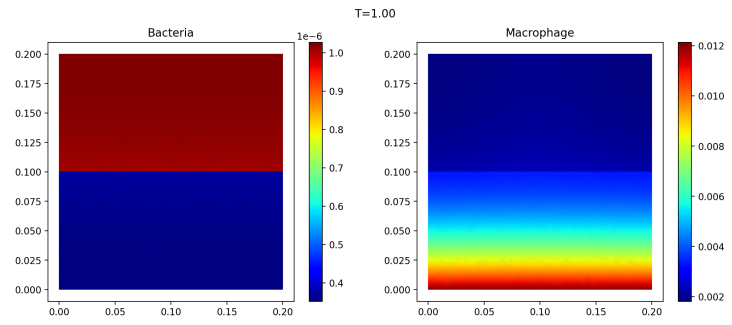


**(c)** No velocity, $t = 1$ day.

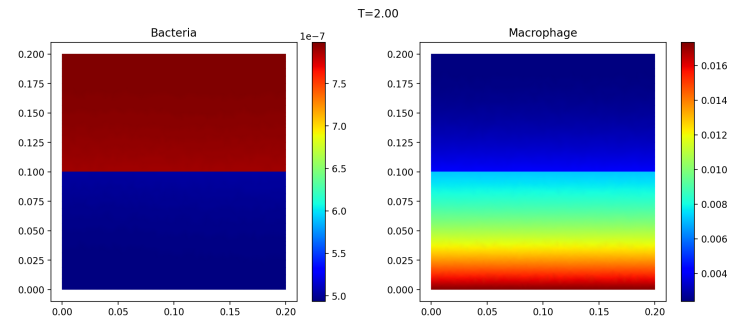


**(d)** No velocity, $t = 2$ day.

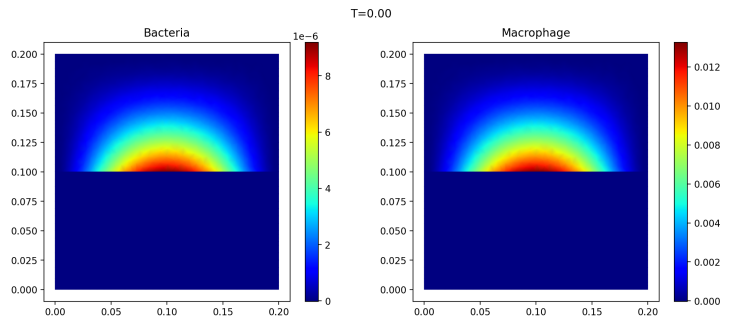


**(e)** With velocity, $t = 0$ day.

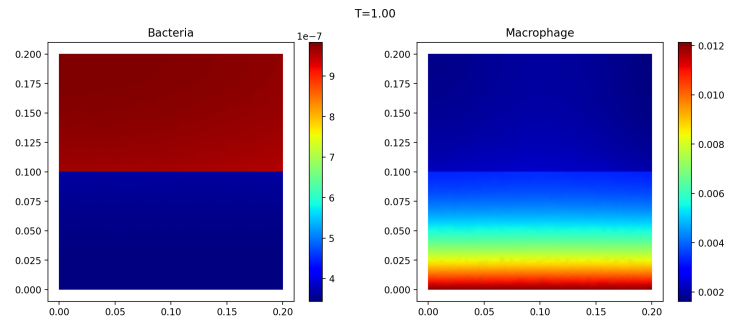


**(f)** With velocity, $t = 1$ day.

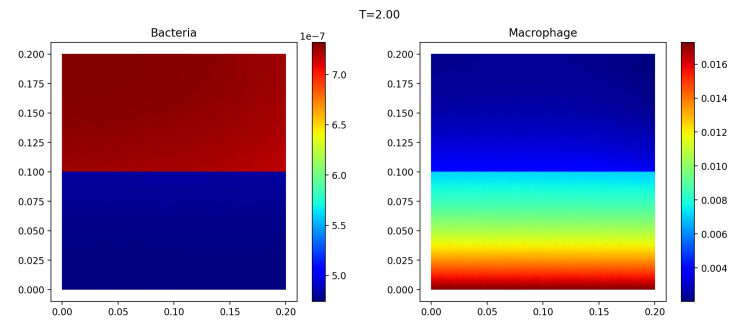


**(g)** With velocity, $t = 2$ day.

**Figure S9:** Evolution of the system for parameter set No. 646, with and without the mucus velocity field. (a) Concentration fitting for bacteria and macrophages, with calibration reference data, mucus-region simulation, and tissue-region simulation shown in each plot. (b–d) Spatial distributions without mucus velocity field driven at $t = 0$, 1, and 2 day. (e–g) Spatial distributions with the mucus velocity field driven at the same time points. Each spatial panel shows bacteria on the left and macrophages on the right.

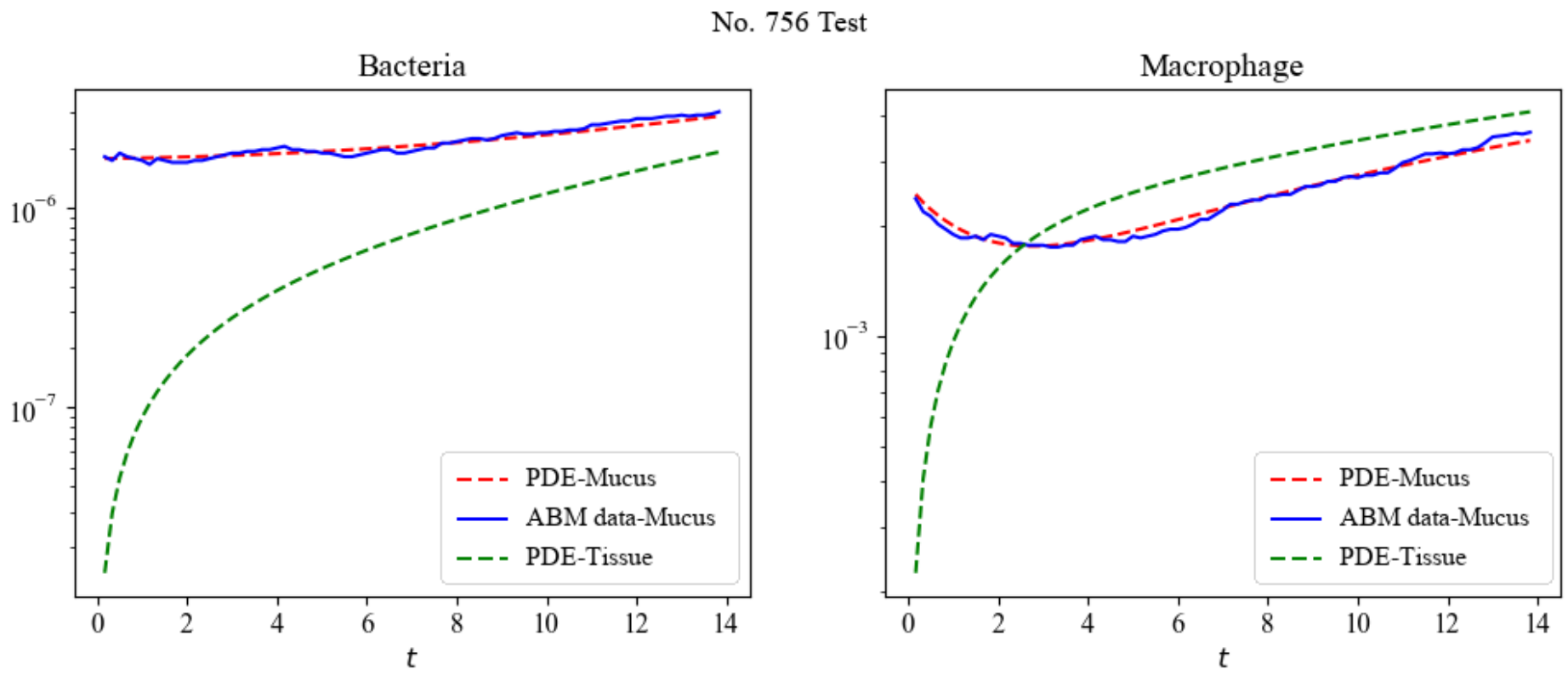


**(a)** Concentration fitting.

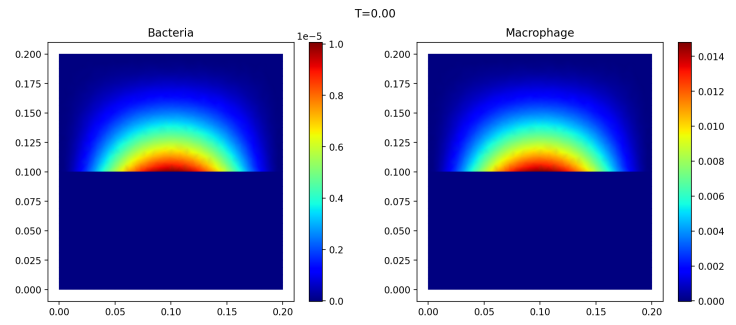

**(b)** No velocity, $t = 0$ day.

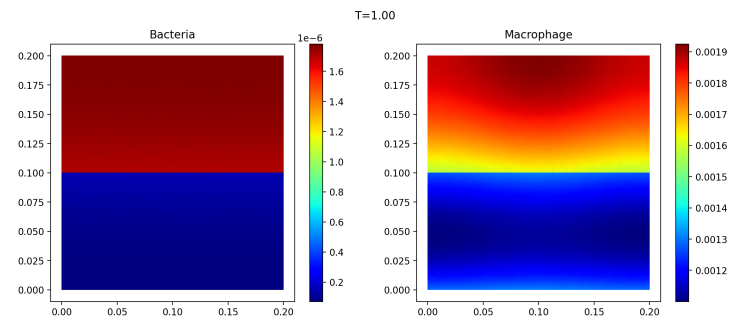

**(c)** No velocity, $t = 1$ day.

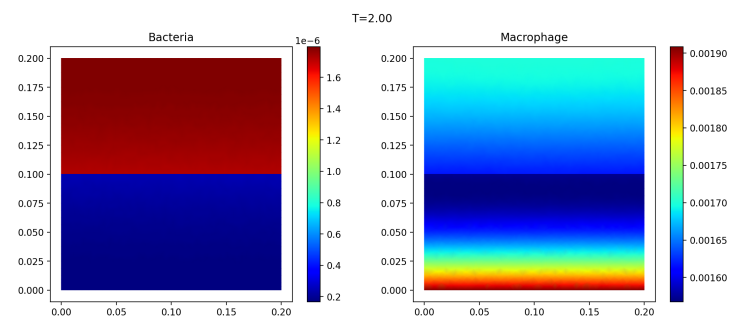

**(d)** No velocity, $t = 2$ day.

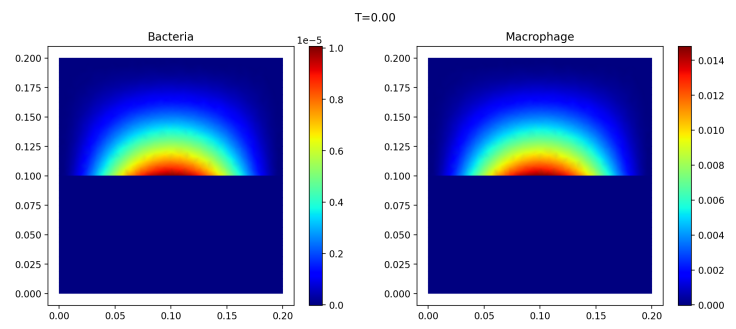

**(e)** With velocity, $t = 0$ day.

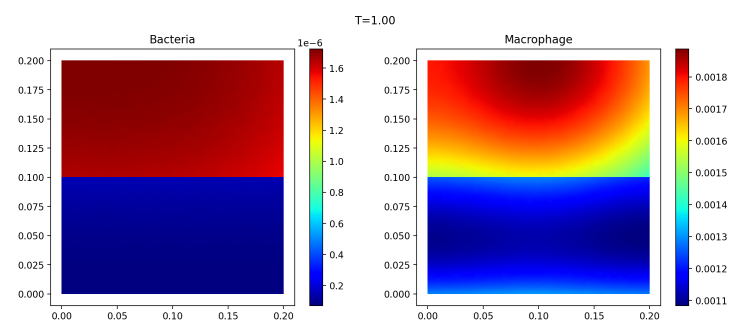

**(f)** With velocity, $t = 1$ day.

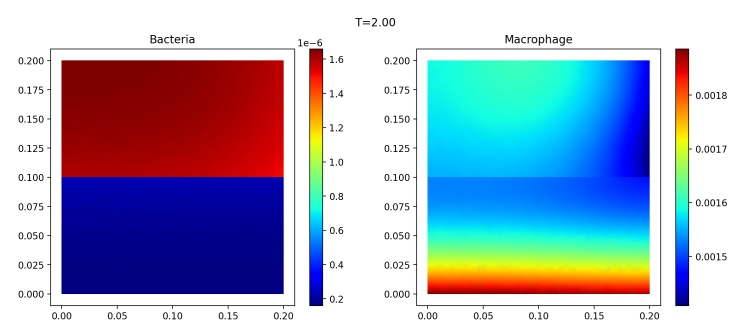

**(g)** With velocity, $t = 2$ day.

**Figure S10:** Evolution of the system for parameter set No. 756, with and without the mucus velocity field. (a) Concentration fitting for bacteria and macrophages, with calibration reference data, mucus-region simulation, and tissue-region simulation shown in each plot. (b–d) Spatial distributions without mucus velocity field driven at $t = 0$, 1, and 2 day. (e–g) Spatial distributions with the mucus velocity field driven at the same time points. Each spatial panel shows bacteria on the left and macrophages on the right.

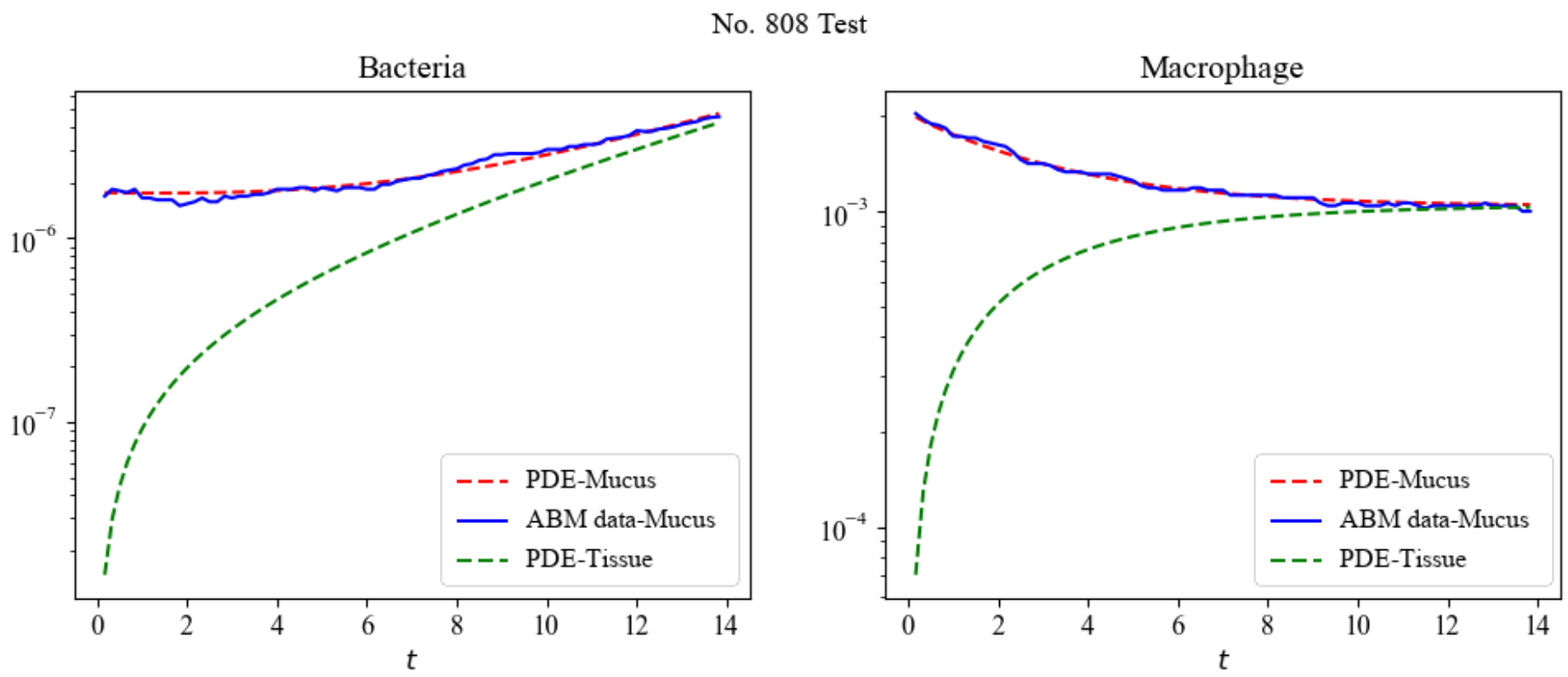


**(a)** Concentration fitting.

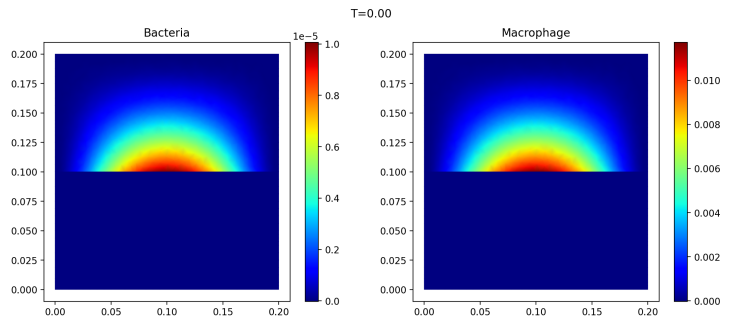

**(b)** No velocity, $t = 0$ day.

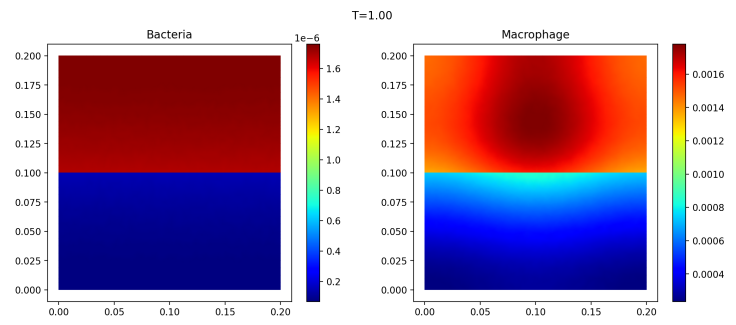

**(c)** No velocity, $t = 1$ day.

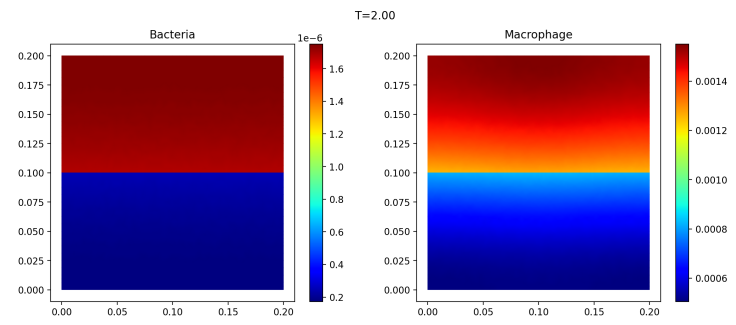

**(d)** No velocity, $t = 2$ day.

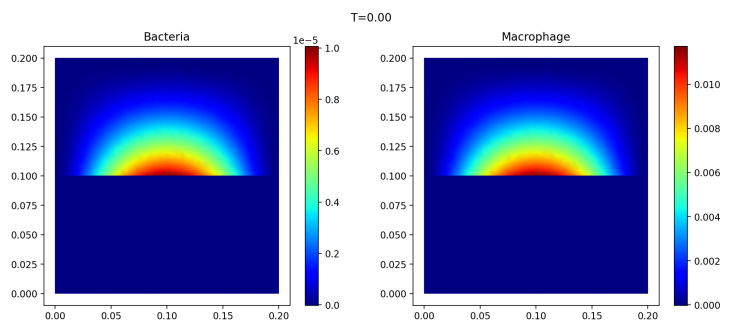

**(e)** With velocity, $t = 0$ day.

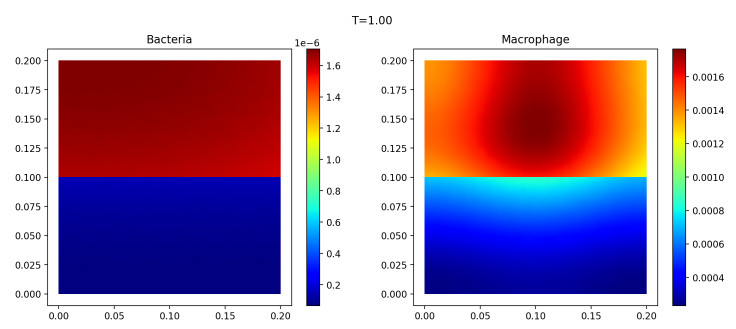

**(f)** With velocity, $t = 1$ day.

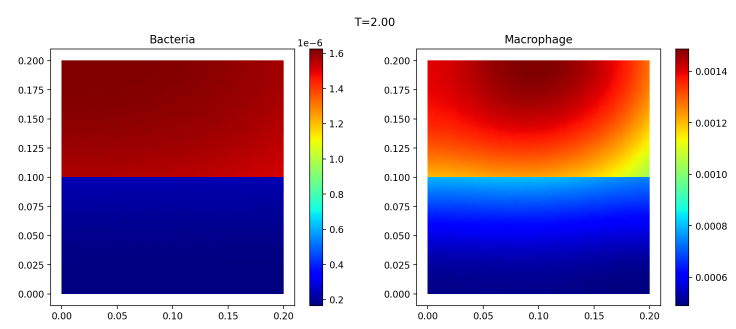

**(g)** With velocity, $t = 2$ day.

**Figure S11:** Evolution of the system for parameter set No. 808, with and without the mucus velocity field. (a) Concentration fitting for bacteria and macrophages, with calibration reference data, mucus-region simulation, and tissue-region simulation shown in each plot. (b–d) Spatial distributions without mucus velocity field driven at $t = 0$, 1, and 2 day. (e–g) Spatial distributions with the mucus velocity field driven at the same time points. Each spatial panel shows bacteria on the left and macrophages on the right.

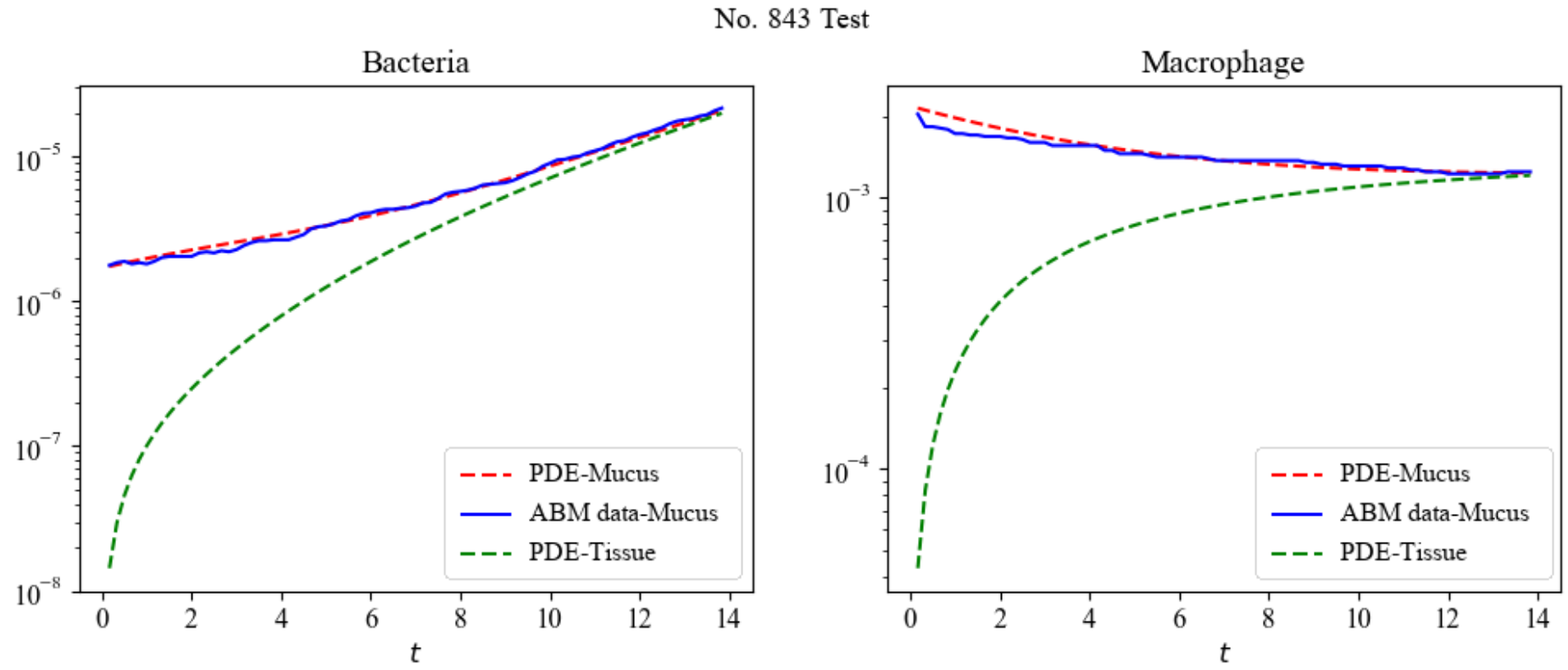


**(a)** Concentration fitting.

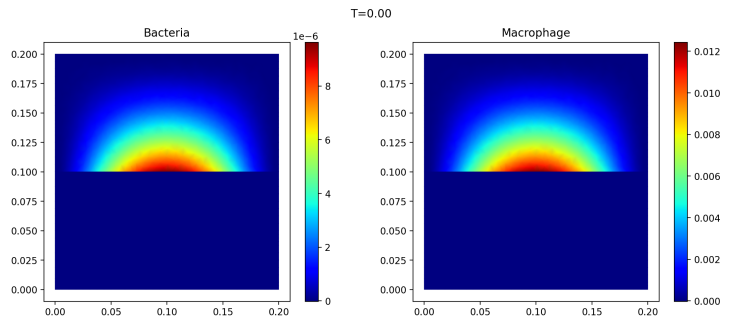


**(b)** No velocity, $t = 0$ day.

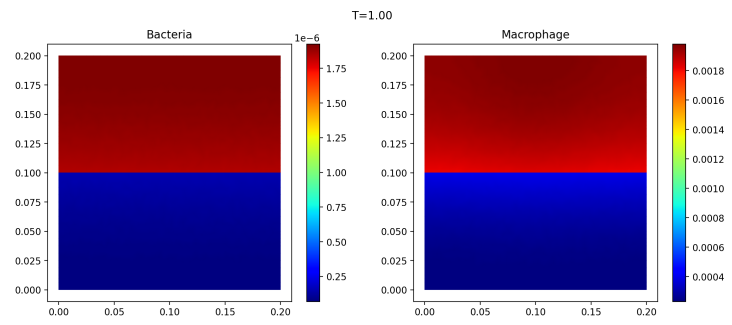


**(c)** No velocity, $t = 1$ day.

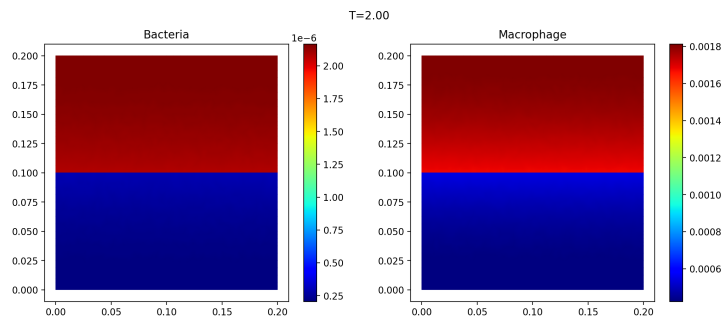


**(d)** No velocity, $t = 2$ day.

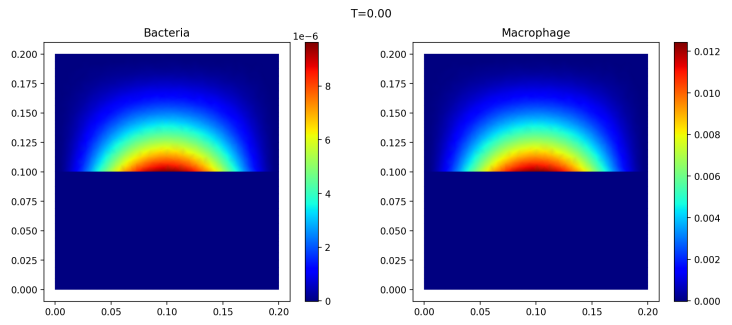


**(e)** With velocity, $t = 0$ day.

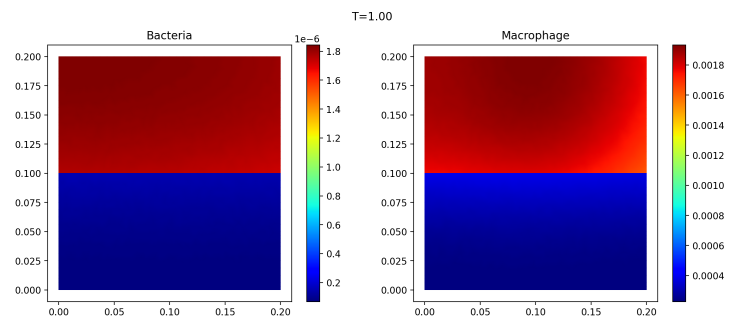


**(f)** With velocity, $t = 1$ day.

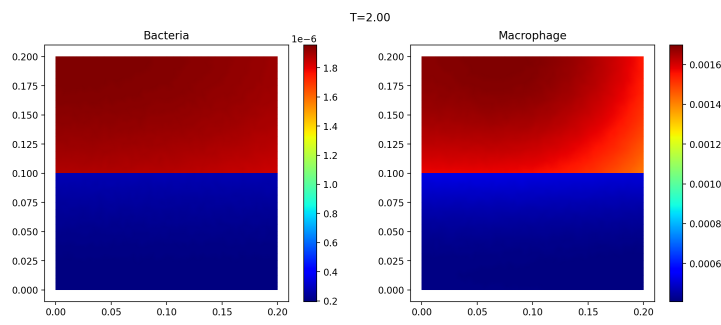


**(g)** With velocity, $t = 2$ day.

**Figure S12:** Evolution of the system for parameter set No. 843, with and without the mucus velocity field. (a) Concentration fitting for bacteria and macrophages, with calibration reference data, mucus-region simulation, and tissue-region simulation shown in each plot. (b–d) Spatial distributions without mucus velocity field driven at $t = 0$, 1, and 2 day. (e–g) Spatial distributions with the mucus velocity field driven at the same time points. Each spatial panel shows bacteria on the left and macrophages on the right.

## S6 Full Sensitivity Analysis Results

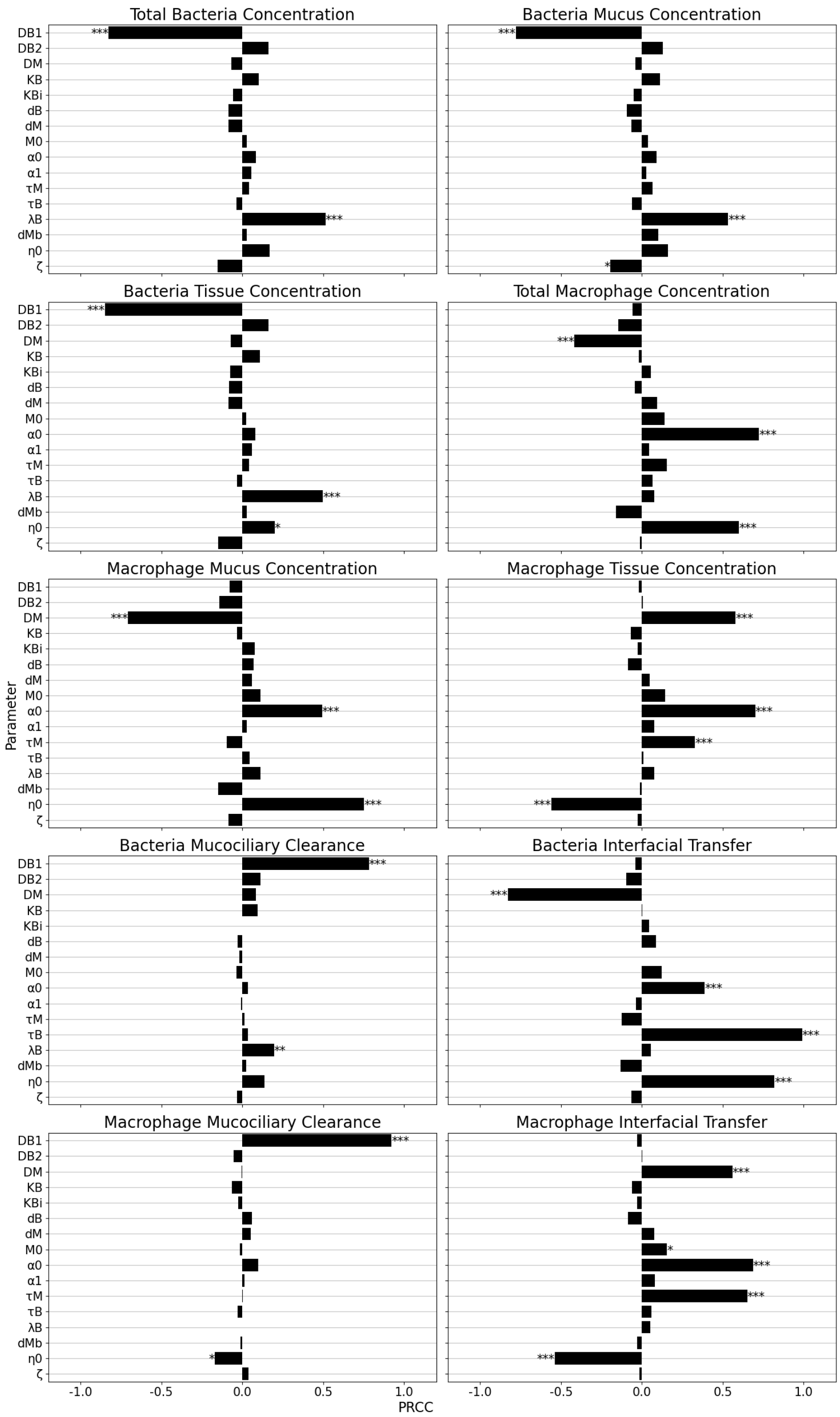


**Figure S13:** Partial rank correlation coefficients (PRCCs) between model parameters and all simulation outputs.

# S7 Function heatmap of antibiotic bacteria killing rate and viscosity $\eta$

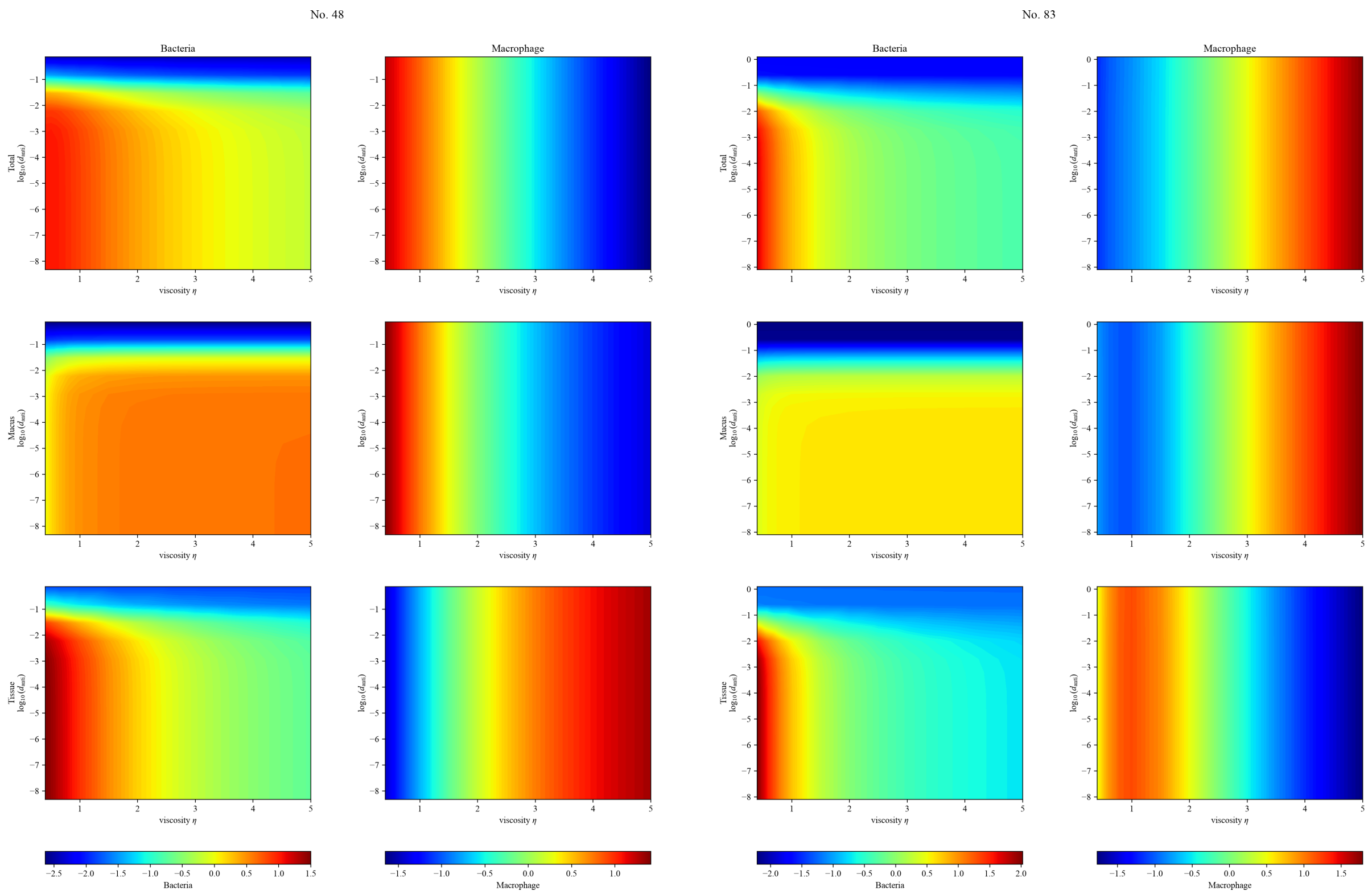


**Figure S14:** Standardized treatment-response heatmaps for parameter sets No. 48 (left) and No. 83 (right). Rows show total, mucus, and tissue responses, and columns show bacteria and macrophages. Mucus and tissue quantities are standardized as $(x - \mu)/\sigma$ using global statistics across all parameter sets; the total response is the sum of the standardized mucus and tissue responses.

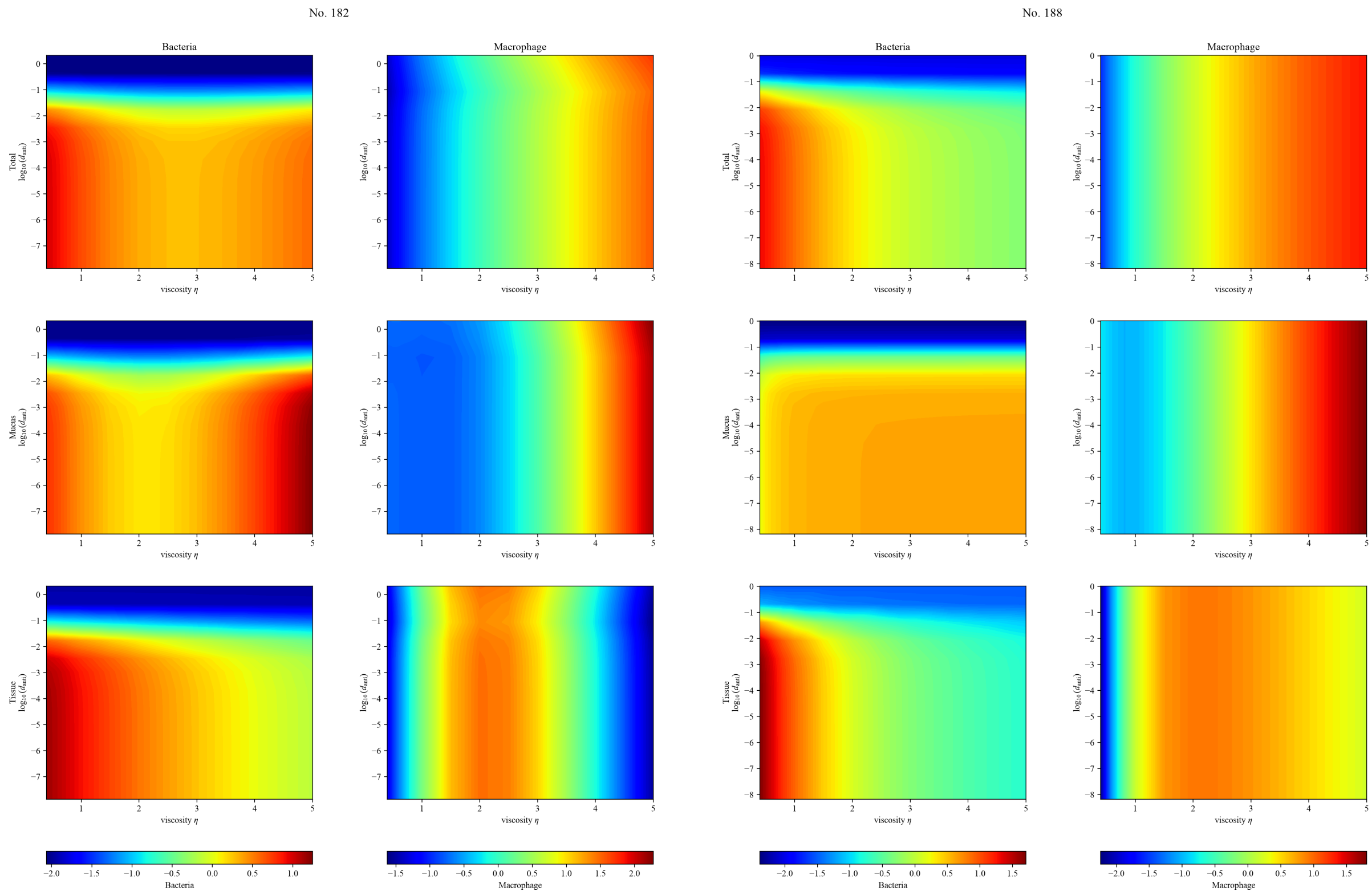


**Figure S15:** Standardized treatment-response heatmaps for parameter sets No. 182 (left) and No. 188 (right). Panel layout, normalization, total-response definition, and color-scale conventions are as described in Fig. S14.

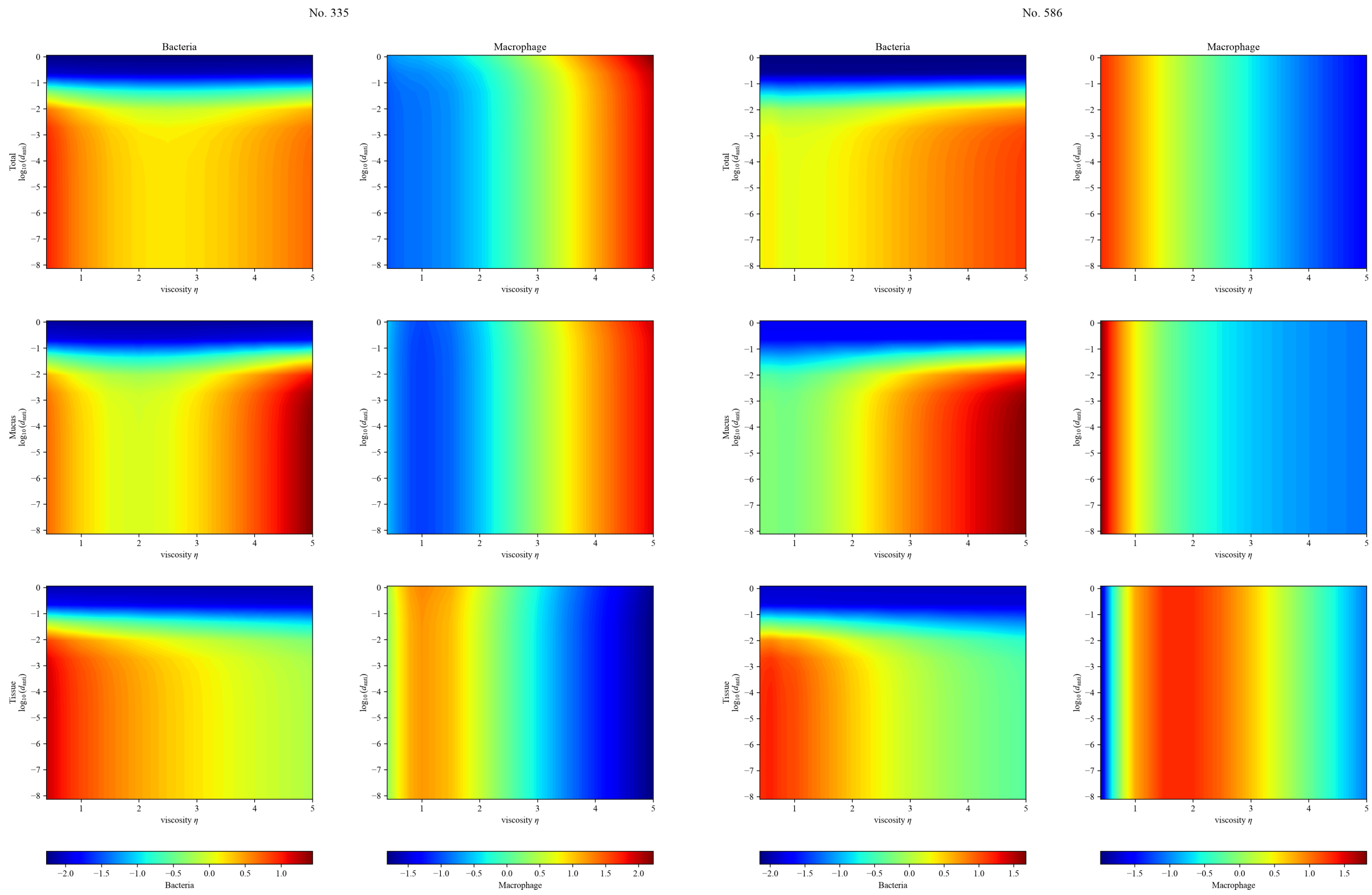


**Figure S16:** Standardized treatment-response heatmaps for parameter sets No. 335 (left) and No. 586 (right). Panel layout, normalization, total-response definition, and color-scale conventions are as described in Fig. S14.

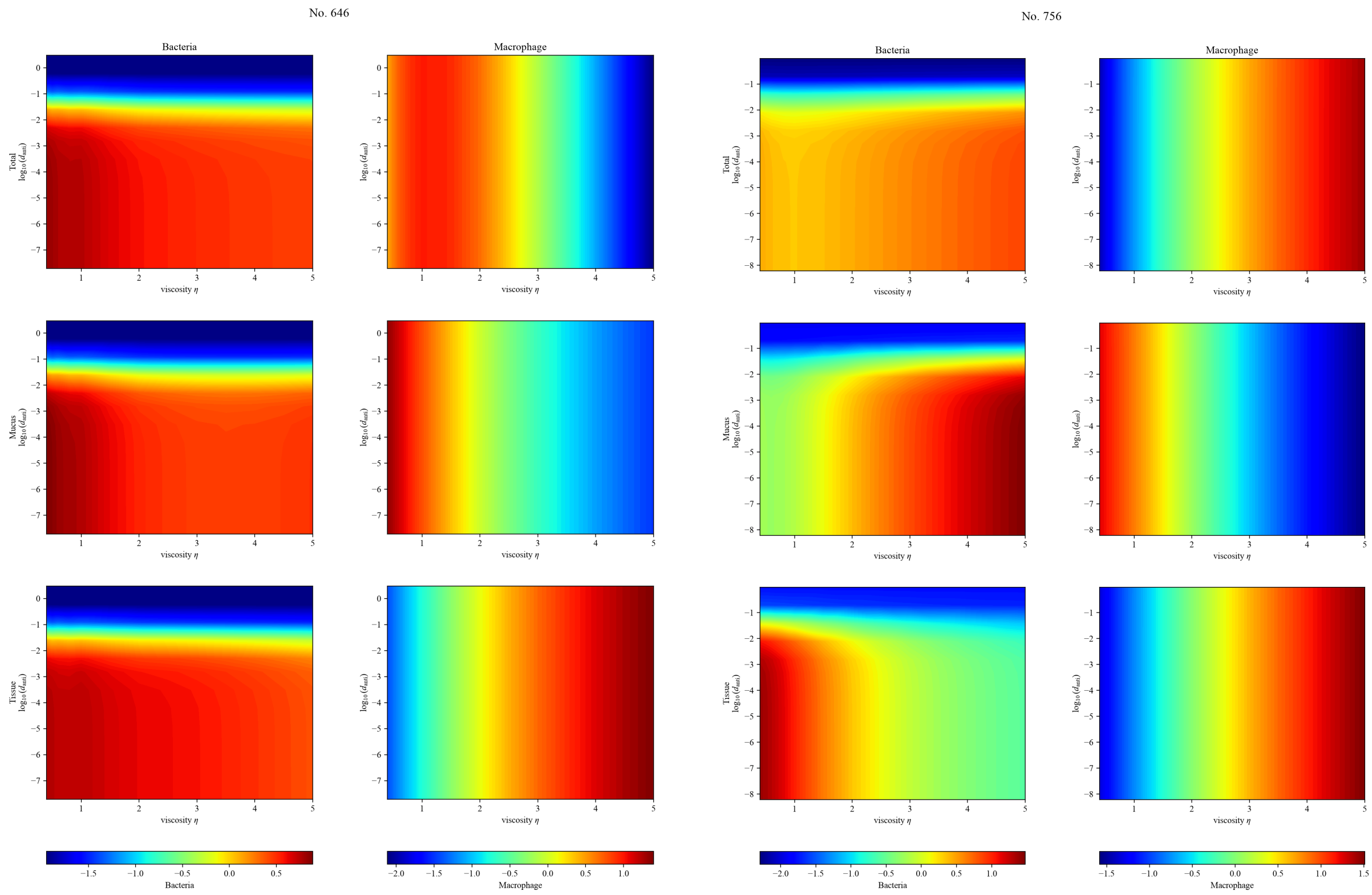


**Figure S17:** Standardized treatment-response heatmaps for parameter sets No. 646 (left) and No. 756 (right). Panel layout, normalization, total-response definition, and color-scale conventions are as described in Fig. S14.

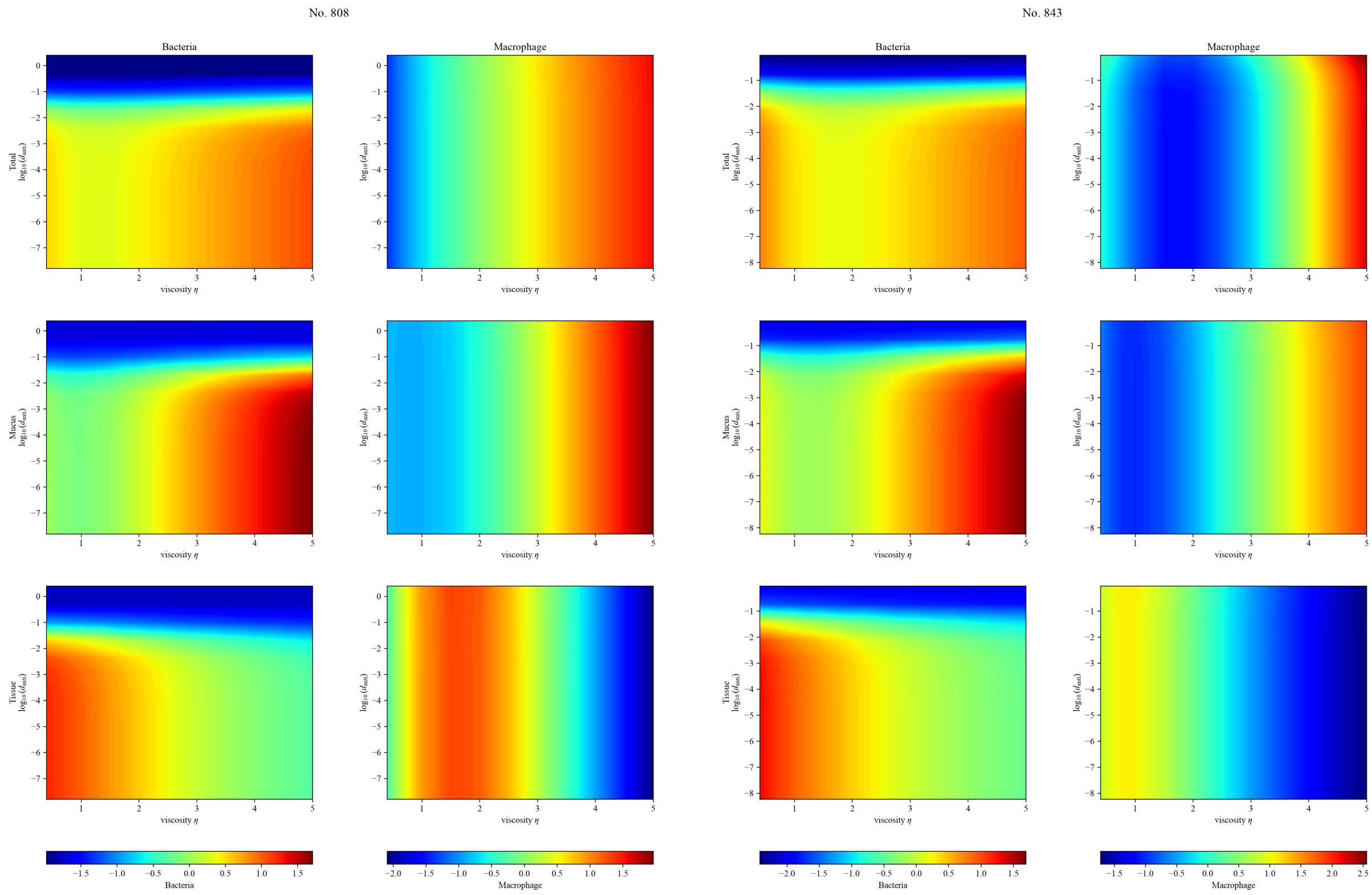


**Figure S18:** Standardized treatment-response heatmaps for parameter sets No. 808 (left) and No. 843 (right). Panel layout, normalization, total-response definition, and color-scale conventions are as described in Fig. S14.

## References


[1] Kenneth N Olivier, David J Weber, Richard J Wallace, Jr, Ali R Faiz, Ji-Hyun Lee, Yansheng Zhang, Barbara A Brown-Elliot, Allison Handler, Rebecca W Wilson, Michael S Schechter, Lloyd J Edwards, Subha Chakraborti, Michael R Knowles, and Nontuberculous Mycobacteria in Cystic Fibrosis Study Group. Nontuberculous mycobacteria. i: multicenter prevalence study in cystic fibrosis. *Am. J. Respir. Crit. Care Med.*, 167(6):828–834, March 2003.

[2] Paul J Planet. Adaptation and evolution of pathogens in the cystic fibrosis lung. *Journal of the Pediatric Infectious Diseases Society*, 11(Supplement_2):S23–S31, 09 2022. ISSN 2048-7207. doi: 10.1093/jpids/piac073. URL `https://doi.org/10.1093/jpids/piac073`.

[3] Devi Jaganathan, Emanuela M. Bruscia, and Benjamin T. Kopp. Emerging concepts in defective macrophage phagocytosis in cystic fibrosis. *International Journal of Molecular Sciences*, 23(14), 2022. ISSN 1422-0067. doi: 10.3390/ijms23147750. URL `https://www.mdpi.com/1422-0067/23/14/7750`.

[4] Manuella Lévêque, Sophie Le Trionnaire, Paola Del Porto, and Corinne Martin-Chouly. The impact of impaired macrophage functions in cystic fibrosis disease progression. *Journal of Cystic Fibrosis*, 16(4):443–453, 2017. ISSN 1569-1993. doi: https://doi.org/10.1016/j.jcf.2016.10.011. URL `https://www.sciencedirect.com/science/article/pii/S1569199316306464`.

[5] Keren B Turton, Rebecca J Ingram, and Miguel A Valvano. Macrophage dysfunction in cystic fibrosis: Nature or nurture? *Journal of Leukocyte Biology*, 109(3):573–582, 07 2020. ISSN 0741-5400. doi: 10.1002/JLB.4RU0620-245R. URL `https://doi.org/10.1002/JLB.4RU0620-245R`.

[6] Beth L Laube, Kathryn A Carson, Christopher M Evans, Vanessa L Richardson, Gail Sharpless, Pamela L Zeitlin, and Peter J Mogayzel, Jr. Changes in mucociliary clearance over time in children with cystic fibrosis. *Pediatr. Pulmonol.*, 55(9):2307–2314, September 2020.

[7] Louis R Joslyn, Jennifer J Linderman, and Denise E Kirschner. A virtual host model of mycobacterium tuberculosis infection identifies early immune events as predictive of infection outcomes. *J. Theor. Biol.*, 539(111042):111042, April 2022.

[8] Michael J Pitcher, Ruth Bowness, Simon Dobson, Raluca Eftimie, and Stephen H Gillespie. Modelling the effects of environmental heterogeneity within the lung on the tuberculosis life-cycle. *J. Theor. Biol.*, 506(110381):110381, December 2020.

[9] Eliezer Flores-Garza, Mario A Zetter, Rogelio Hernández-Pando, and Elisa Domínguez-Hüttinger. Mathematical model of the immunopathological progression of tuberculosis. *Front. Syst. Biol.*, 2, August 2022.

[10] Wenjing Zhang. Deterministic and stochastic in-host tuberculosis models for bacterium-directed and host-directed therapy combination. *Math. Med. Biol.*, 39(2):126–155, June 2022.

[11] Catherine Weathered, Kelly Pennington, Patricio Escalante, and Elsje Pienaar. The role of biofilms, bacterial phenotypes, and innate immune response in mycobacterium avium colonization to infection. *Journal of Theoretical Biology*, 534:110949, 2022.

[12] Catherine Weathered, Ning Wei, and Elsje Pienaar. Reduced macrophage killing of m. avium drives infection risk in post-menopausal patients. *Tuberculosis (Edinb.)*, 139(102304): 102304, March 2023.

[13] Caitlin Hult, Joshua T Mattila, Hannah P Gideon, Jennifer J Linderman, and Denise E Kirschner. Neutrophil dynamics affect mycobacterium tuberculosis granuloma outcomes and dissemination. *Front. Immunol.*, 12:712457, October 2021.

[14] Jess A Millar, J Russell Butler, Stephanie Evans, Joshua T Mattila, Jennifer J Linderman, Joanne L Flynn, and Denise E Kirschner. Spatial organization and recruitment of non-specific T cells may limit T cell-macrophage interactions within mycobacterium tuberculosis granulomas. *Front. Immunol.*, 11:613638, 2020.

[15] Wenrui Hao, Larry S Schlesinger, and Avner Friedman. Modeling granulomas in response to infection in the lung. *PLoS One*, 11(3):e0148738, 2016.

[16] Wenrui Hao, Elliott D Crouser, and Avner Friedman. Mathematical model of sarcoidosis. *Proceedings of the National Academy of Sciences*, 111(45):16065–16070, 2014.

[17] Blake A Bartlett, Yu Feng, Catherine A Fromen, and Ashlee N Ford Versypt. Computational fluid dynamics modeling of aerosol particle transport through lung airway mucosa. *Comput. Chem. Eng.*, 179(108458):108458, November 2023.

[18] Ling Xu, Pejman Senaei, and Yi Jiang. Modeling tissue-scale ciliary transport and mixing in three-dimensional newtonian flow. *PLoS Comput. Biol.*, 21(12):e1012766, December 2025.

[19] Catherine Weathered, Kelly Pennington, Patricio Escalante, and Elsje Pienaar. Agent-based model indicates chemoattractant signaling caused by mycobacterium avium biofilms in the lung airway increases bacterial loads by spatially diverting macrophages. *Tuberculosis (Edinb.)*, 138(102300):102300, January 2023.

[20] F Krombach, S Münzing, A M Allmeling, J T Gerlach, J Behr, and M Dörger. Cell size of alveolar macrophages: an interspecies comparison. *Environ. Health Perspect.*, 105 Suppl 5: 1261–1263, September 1997.

[21] M Loferer-Krössbacher, J Klima, and R Psenner. Determination of bacterial cell dry mass by transmission electron microscopy and densitometric image analysis. *Appl. Environ. Microbiol.*, 64(2):688–694, February 1998.

[22] A Signore, C D'Alessandria, E Lazzeri, and R Dierckx. Can we produce an image of bacteria with radiopharmaceuticals? *Eur. J. Nucl. Med. Mol. Imaging*, 35(6):1051–1055, June 2008.

[23] Edmund Jones, David Epstein, and Leticia García-Mochón. A procedure for deriving formulas to convert transition rates to probabilities for multistate markov models. *Medical Decision Making*, 37(7):779–789, 2017.

[24] Emma Denneny, Jagdeep Sahota, Richard Beatson, David Thornton, Joy Burchell, and Joanna Porter. Mucins and their receptors in chronic lung disease. *Clinical & translational immunology*, 9(3):e01120, 2020.

[25] Arindam Fadikar, Dave Higdon, Jiangzhuo Chen, Bryan Lewis, Srinivasan Venkatramanan, and Madhav Marathe. Calibrating a stochastic, agent-based model using quantile-based emulation. *SIAM/ASA Journal on Uncertainty Quantification*, 6(4):1685–1706, 2018.

[26] Simeone Marino, Ian B. Hogue, Christian J. Ray, and Denise E. Kirschner. A methodology for performing global uncertainty and sensitivity analysis in systems biology. *Journal of Theoretical Biology*, 254(1):178–196, 2008. ISSN 0022-5193. doi: https://doi.org/10.1016/j.jtbi.2008.04.011. URL https://www.sciencedirect.com/science/article/pii/S0022519308001896.

[27] Stacey L Martiniano, Jerry A Nick, and Charles L Daley. Nontuberculous mycobacterial infections in cystic fibrosis. *Clin. Chest Med.*, 43(4):697–716, December 2022.

[28] Naif Sulaiman, Beatriz Martins, Diana Moreira-Sousa, Ana Aguiar, John R Hurst, James Brown, Raquel Duarte, and Marc Lipman. Optimising non-pharmacological interventions in people with non-tuberculous mycobacterial pulmonary disease: a systematic review. *ERJ Open Res.*, 11(6):533–2025, November 2025.

[29] Victor E Ortega, Elsje Pienaar, and Patricio Escalante. Genetic susceptibility to nontuberculous mycobacterial pulmonary disease: Is it the chicken or the egg? *Am. J. Respir. Crit. Care Med.*, 211(11):2150–2152, November 2025.

[30] Kyungtaek Park, Hyejin Kim, Hee Jae Huh, Ho Namkoong, Naoki Hasegawa, Tomoyasu Nishimura, Takanori Asakura, Kozo Morimoto, Atsuyuki Kurashima, Yosuke Omae, Yosuke Kawai, Katsushi Tokunaga, Joanne Berghout, Kevin P Fennelly, Steven M Holland, Jaeyoung Cho, Jae-Joon Yim, Sungho Won, Byung Woo Jhun, and Hong-Hee Won. Novel genetic loci for nontuberculous mycobacterial pulmonary disease and potential protective effect of body mass index. *Am. J. Respir. Crit. Care Med.*, 211(11):2191–2202, November 2025.

[31] Pierre-Régis Burgel, Kevin W Southern, Charlotte Addy, Alberto Battezzati, Claire Berry, Jean-Philippe Bouchara, Edwin Brokaar, Whitney Brown, Pilar Azevedo, Isabelle Durieu, Miquel Ekkelenkamp, Felicity Finlayson, Julian Forton, Johanna Gardecki, Pavla Hodkova, Gina Hong, Jacqueline Lowdon, Su Madge, Clémence Martin, Edward McKone, Anne Munck, Chee Y Ooi, Lucy Perrem, Amanda Piper, Andrew Prayle, Felix Ratjen, Margaret Rosenfeld, Don B Sanders, Carsten Schwarz, Giovanni Taccetti, Claire Wainwright, Natalie E West, Michael Wilschanski, Amanda Bevan, Carlo Castellani, Pavel Drevinek, Silvia Gartner, Andrea Gramegna, Elise Lammertyn, Eddie Edwina C Landau, Barry J Plant, Alan R Smyth, Silke van Koningsbruggen-Rietschel, and Peter G Middleton. Standards for the care of people with cystic fibrosis (CF); recognising and addressing CF health issues. *J. Cyst. Fibros.*, 23(2):187–202, March 2024.

[32] Nicholas A Cilfone, Denise E Kirschner, and Jennifer J Linderman. Strategies for efficient numerical implementation of hybrid multi-scale agent-based models to describe biological systems. *Cellular and molecular bioengineering*, 8(1):119–136, 2015.

[33] Winston Garira. A primer on multiscale modelling of infectious disease systems. *Infectious Disease Modelling*, 3:176–191, 2018.

[34] Yoram Vodovotz and Gary An. Agent-based models of inflammation in translational systems biology: A decade later. *Wiley Interdisciplinary Reviews: Systems Biology and Medicine*, 11(6):e1460, 2019.
